# Antagonizing Il10 and Il4 signaling via intracerebral decoy receptor expression attenuates Aβ accumulation

**DOI:** 10.1186/s40478-025-01968-3

**Published:** 2025-03-07

**Authors:** Emily J. Koller, Karen N. McFarland, Conner Angelle, John Howard, Danny Ryu, Kristy D. Dillon, Aya Erquizi, Mihir Beheray, Elsa Gonzalez De La Cruz, Pedro E. Cruz, Jada Lewis, Todd E. Golde, Yona Levites, Paramita Chakrabarty

**Affiliations:** 1 Department of Neuroscience, University of Florida, Gainesville, FL-32610 USA; 2 Center for Translational Research in Neurodegenerative Disease, University of Florida, Gainesville, FL-32610 USA; 3 Department of Pharmacology and Chemical Biology, School of Medicine, Emory University, Atlanta, GA USA; 4 Goizueta Brain Health Institute and Alzheimer’s Disease Research Center, Emory University School of Medicine, Atlanta, GA USA; 5 Department of Physiological Sciences, University of Florida, Gainesville, FL-32610 USA; 6 Present Address: Medpace, 2120 West Walnut Hill Lane, Irving, TX 75038 USA; 7 Present Address: Department of Pathology, University of Florida, Gainesville, FL-32610 USA

**Keywords:** Soluble receptor, Il10, Il4, Amyloid, Tau, Therapy, Circadian rhythm

## Abstract

Multiple lines of evidence indicate that immune signaling can impact the pathological progression in Alzheimer’s disease (AD), including amyloid deposition, tau aggregation, synaptic pathology and neurodegenerative trajectory. In earlier studies, we reported that intracerebral expression of the anti-inflammatory cytokines, Interleukin-10 (Il10) and Interleukin-4 (Il4), increased amyloid β (Aβ) burden in TgCRND8 mice, a preclinical model of AD-type amyloidosis. As both Interleukin-10 receptor (IL10R) and Interleukin-4 receptor (IL4R) are upregulated in an age-progressive manner in rodent models of AD and in specific regions of human AD brains, we hypothesized that a decoy receptor strategy specifically targeting Il10 and Il4 signaling could have a disease-modifying effect. We derivatized the ectodomains of mouse Il10R (sIl10R) and mouse Il4R (sIl4R) into corresponding recombinant solubilized receptor forms and delivered these intracranially into neonatal TgCRND8 mice or hippocampally into adult TgCRND8 mice with pre-existing Aβ deposits. AAV-mediated expression of sIl10R and sIl4R robustly attenuated Aβ burden in TgCRND8 mice when expressed neonatally while in the hippocampus injection cohort, AAV-sIl4R, but not sIl10R, reduced Aβ burden. sIl10R and sIl4R had opposing effects on microglial and astrocyte proliferation, with sIl10R generally reducing gliosis. RNAseq analysis showed that sIl10R likely acts as a microglial immune checkpoint inhibitor while both sIl10R and sIl4R expression show unexpected impacts on genes related to circadian rhythm. Notably, neither Il10 nor sIl10R expression altered tau pathology in two tau transgenic models, despite robust expression and impacts on glial proliferation. Together, these data reveal that decoy receptor mediated targeting of physiological Il10 or Il4 signaling can beneficially impact amyloid deposition and thus represent novel immunomodulatory approaches for AD therapy.

**Supplementary Information:**

The online version contains supplementary material available at 10.1186/s40478-025-01968-3.

## Background

The involvement of the immune system preceding and accompanying the development of Alzheimer’s disease (AD) is now widely accepted. In addition to evidence from neuroimaging studies [1–5], genetic evidence supporting this idea came from the discovery of immune gene variants, albeit rare, that modify the risk of AD [6]. In addition, several candidate AD-causal gene variants specific to neurons or oligodendrocytes have been identified, which may also affect the function of the immune cells in a non-cell autonomous manner. Recently available massive-level genome wide association meta-studies that were integrated with expression quantitative trait loci data show that at least 51% of AD-causal risk loci contain myeloid or microglial cell-specific genes [7, 8]. A promising study integrating AD endophenotypes with polygenic risk score approach at the individual patient level has revealed the early role of immune cell dysfunction in AD pathogenesis [5]. Overall, these findings validate the hypothesis that immune function is mechanistically related to AD etiology through multiple mechanisms.

The function of many of the AD-risk variants of immune genes (*TREM2*, *ABCA7*, *PLCG2*, *ABI3*) is to suppress microglial phagocytotic clearance of cellular debris, which could also extend to amyloid β (Aβ) and apoptotic cells [9]. However, in preclinical studies that investigated each of the causal immune gene variants in transgenic models of *APP*/Aβ and tau, the mechanisms have been less clear [10, 11]. While some studies report that suppressing microglial function reduces the pathological burden of AD-associated Aβ and tau [12–14], others have shown that early glial activation mediated by cytokines could have a beneficial effect in preventing or clearing protein aggregates [15–17]. Indeed, we have shown previously that intracerebral overexpression of cytokines with canonical anti-inflammatory properties, Il4 and Il10, increased Aβ deposition [18, 19] whereas, we and others have observed the opposite with multiple canonical proinflammatory cytokines [15, 16, 20–23].

Decoy receptors are one of the various mechanisms that have been naturally found to negatively regulate cytokine function [24]. These receptors typically bind their cognate cytokines with high affinity, but they are unable to transduce the signals to downstream partners as they lack their cytoplasmic domains, thus blunting the cellular outcome and in many cases, scavenging the cytokines from further activity. For example, soluble forms of ILR2 (sILR2) forms complexes with both pro-forms and mature forms of IL-1α and IL-1β, directly regulating IL-1 function [25]. Expression of Decoy receptor 3 (DcR3), a soluble protein that can neutralize TNF-family members, was shown to be protective against AD-amyloidosis [26]. Decoy receptors against specific cytokines could be potentially used to block or scavenge selected ligands [27], thus acting as potential immune checkpoint inhibitors.

Based on our earlier studies that Il10 and Il4 worsen AD-amyloidosis [18, 19], we reasoned that blocking the action of these cytokines within the brain could have a protective effect by acting as immune checkpoint inhibitors and preventing Aβ deposition. As opposed to overexpression of activating cytokines, inhibiting the action of these anti-inflammatory cytokines within the brain represents a more physiologic blockade, and also avoids potential impacts of non-targeted whole-body inhibition of these pathways [28, 29]. Towards this, we expressed the ectodomains of Il10rα (soluble or sIl10r) or Il4rα (soluble or sIl4r) in the brains of neonatal or adult human mutant *APP* transgenic TgCRND8 mice using adeno-associated viruses (AAV). Intracerebral expression AAV-sIl4R attenuated Aβ deposition in both cohorts of TgCRND8 mice, while sIl10R was more effective in the neonatal cohort. Additionally, we confirmed that the Il10/sIl10R pathway does not influence the neuropathology in transgenic models of tau. Overall, our results indicate that immune modifying therapies targeting specific cytokines have context-dependent effects as an anti-amyloid therapeutic.

## Methods

### Mice

Experiments were approved by the Institutional Animal Care and Use Committee at the University of Florida and Emory University. The TgCRND8 mice overexpressing amyloid precursor protein (*APP*) begin to develop Thioflavin-S positive Aβ amyloid plaques by 3 months of age and impaired performance in a fear-conditioning cognitive task by 6 months of age. TgCRND8 mice were bred in house and housed three to five to a cage and maintained on ad libitum food and water with a 12 h light/dark cycle. We used two *MAPT* transgenic models maintained in-house– the rTg4510 [30] and the JNPL3 models [31]. The rTg4510 model develops cortical and hippocampal degeneration within 6-months of age whereas the JNPL3 mice (preferentially females) develop hindlimb paralysis correlated with tau aggregation in the spinal cord and brain.

### AAV preparation

Recombinant AAV packaged in serotype 1 expressing sIl10R and sIl4R under the control of the cytomegalovirus enhancer/chicken β actin promoter (CBA) were generated as described previously [32]. Briefly, AAV vectors containing the transgene, promoter, WPRE and bovine growth hormone polyA were transfected, along with helper plasmids, in HEK293T cells. 48 h after transfection, cells were harvested and lysed in the presence of 0.5% Sodium Deoxycholate and 50U/ml Benzonase (Sigma) by freeze thawing, and the virus isolated using a discontinuous Iodixanol gradient followed by concentrating using Amicon Ultra filter 100,000 MWCO (Millipore). The genomic titer of AAV was determined by quantitative PCR.

### Neonatal injections of recombinant AAV

Intracerebroventricular (ICV) injections of recombinant AAV were carried out on neonatal day P0 or P2 as described previously [32]. Recombinant AAV serotype 1 (10^13^ genomes/ml) encoding sIl10R, sIl4R, or GFP from the CBA promoter were administrated bilaterally into each cerebral ventricle (2 µL/hemisphere). Using this strategy, these soluble receptors were predominantly expressed within the neurons followed by secretion in the parenchyma. An additional cohort of control non-injected mice was also added to the study. Mice were aged 3-months and euthanized. Brains were harvested and left hemibrain was fixed overnight in 4% paraformaldehyde at 4 °C followed by processing and paraffin embedding. The right hemibrain was snap frozen and then stored at -80 °C.

### Hippocampal injections of recombinant AAV

6-months old TgCRND8 mice with preexisting amyloid pathology were injected stereotaxically with recombinant AAV1 (10^13^ genomes/ml) encoding sIl10R, sIl4R, or GFP in the hippocampus using coordinates from Bregma: A/*P* − 2.2, L +/−1.6, D/V − 1.2. The AAVs were administrated bilaterally into each hippocampus (2 µL/hemisphere) at a rate of 0.2 µL per min. An additional control cohort was injected with sterile PBS in the hippocampus. At 9-months of age, mice were euthanized and brains processed as following: one hemibrain was cut at the injection site and embedded coronally, while the other hemibrain was freshly dissected to isolate frontal cortex and hippocampus within 2 mm around injection site and snap frozen in isopentane on dry ice for further biochemical analysis.

### Biochemical assay for Aβ and immunoblotting following sequential extraction of brain tissue

Frozen brains (150 mg/mL) were sequentially extracted with RIPA buffer, 2% SDS, and 70% formic acid (FA) containing protease inhibitor cocktail (Roche, Basel, Switzerland) as described previously [18, 19]. Aβ levels from these three sequentially extracted cellular fractions were biochemically quantified using end-specific sandwich ELISA. Aβ40 was captured with mAb 13.1.1 (human Aβ35–40 specific; T.E. Golde) and detected by HRP-conjugated mAb 33.1.1 (human Aβ1–16; T.E. Golde). Aβ42 was captured with mAb 2.1.3 (human Aβ35–42 specific; T.E. Golde) and detected by HRP-conjugated mAb 33.1.1 (human Aβ1–16; T.E. Golde). ELISA results were analyzed using SoftMax Pro software (Molecular Devices, San Jose, CA).

15 µg of RIPA lysate was separated on 4–20% Tris-Glycine gel (Novex, Invitrogen) and transferred to the Immobilon-FL PVDF membrane (MilliporeSigma). Membranes were blocked in 0.5% casein incubated with primary antibodies overnight at 4 °C (GFAP antibody: 1:1000, Cell Signaling; Iba-1 antibody: Dako, 1:1000; β-actin antibody: 1:2000, Proteintech; β-actin antibody: 1:1000, Sigma; GAPDH: 1:1000, Sigma) and protein bands were visualized using the Li-Cor Odyssey M Infrared Imaging system (Li-Cor Biosciences, Lincoln, NE, USA). Relative band intensity was quantified using ImageJ software (National Institutes of Health).

### Immunohistochemistry and thioflavin S histology

5 µm thick formalin-fixed paraffin embedded sections were used for all histology and immunohistochemistry studies. Thioflavin S tissue staining methods were followed as previously described [32]. Paraffin embedded sections were immunohistochemically stained with the biotinylated pan-Aβ antibody Ab5 (1:500; T.E.G.), 33.1.1 antibody (1:5000; T.E.G), Iba-1 antibody (1:1000; Wako), GFAP antibody (1:1000; Cell Signaling), Cd68 antibody (1:1000, Invitrogen), Tmem119 antibody (1:1000, Cell Signaling), Tmem119 antibody (1:2000, Synaptic Systems), V5 antibody (1:1000; Sigma) or FLAG antibody (1:1000; Sigma-Aldrich Inc, St. Louis, IL) followed by incubation in ImmPRESS Polymer Detection Reagent (Vector Laboratories, Burlingame, CA) and color development in 3,3’-diaminobenzidine (DAB) substrate (Vector Laboratories, Burlingame, CA). Slides counterstained with hematoxylin were scanned by Aperio XT System (Leica Biosystems, Buffalo Grove, IL) and analyzed using the ImageScope program. In brief, at least three sections per sample, at least 30 μm apart, were imaged and plaque burden was quantified. For neonatal sIl4R cohort, plaque burden is indicated by actual number of amyloid deposits, whereas for the neonatal sIl10R and hippocampal sIl10R and sIl4R cohorts, plaque burden is indicated by intensity of immunostaining. For Thioflavin S quantification, one section per sample was used by a blinded observer to calculate the number of cored plaques per area using ImageJ. Aβ plaque number was calculated by two independent observers and averages used.

### RNA extraction, RNA sequencing, NanoString codeset and data analysis

#### RNA extraction and sequencing

Brain hemispheres were pulverized under liquid nitrogen and RNA was extracted from pulverized hemi-brain of mice expressing sIl10R, sIl4R or non-injected control (*n* = 4–6) using Trizol (Invitrogen, Waltham, MA) followed by a clean-up step using the RNeasy mini extraction kit with on-column DNase treatment (QIAGEN, Hilden, Germany). A second-round of DNase treatment (TURBO DNA-free kit, Ambion) was employed to remove any residual. RNA. RNA quality was determined with the Qubit RNA HS assay, and analyzed on a Eukaryote Total RNA Nano chip using Agilent Bioanalyzer 2100 (Agilent Technologies, Santa Clara, CA). One microgram of total RNA was used to generate sequencing libraries using the Illumina TruSeq RNA Library Prep kit v2 which enriches for polyA tailed RNA. Libraries were sequenced on paired-end, 100 bp runs on the Nextseq 500 (Illumina, San Diego, CA) using a pooling strategy to minimize batch effects from extraction, library preparation, and sequencing.

#### FASTQ alignment, gene counts, and differential expression analysis

FASTQ files were aligned against the mouse genome (GRCm39) and GRCm39.107 annotation using STAR [33] to generate BAM files. Gene counts were generated from BAM files using Rsamtools (https://bioconductor.org/packages/release/bioc/html/Rsamtools.html) and the “summarizeOverlaps” function with the GenomicAlignments package v1.40.0 [34]. Differential gene expression (DEG) analysis was performed with DESeq2 package v1.44.0 using the “DESeq” function with default settings [35] which fits a generalized linear model for each gene. Subsequent Wald test *P*-values are adjusted for multiple comparisons using the Benjamini–Hochberg method (adjusted *P*-value, padj). Genes with a mean count of less than 10 were removed from further analysis. Pair-wise changes in gene expression levels between groups (sIl10R versus uninjected mice or sIl4R versus uninjected mice) were used to identify DEGs. DEGs were defined as an absolute log_2_ fold change ≥ 0.5 and an adjusted *P*-value ≤ 0.05. Genes with an adjusted P-value of ≤ 0.05 in either sIl10R vs. control or sIl4R vs. control comparisons were included in the Pearson correlation analysis and plot. Cell type signatures and functional pathway annotation using enrichment of DEGs were performed as previously described using goseq version 1.56.0 [36]. Graphs were generated with the tidyverse package version 2.0.0 in R version 4.4.1.

#### Targeted transcriptomics with Nanostring codesets

Transcriptomic analysis was done using a custom NanoString codeset reported earlier [19] and a mouse Neuroinflammation codeset following the manufacturer’s instructions. RCC files from both codesets were imported into nSolver 4.0. Differential gene expression was determined from the raw count data using the DESeq2 package v1.44.0 as described above. Functional pathway analysis and cell type expression changes were conducted as described in the preceding section.

#### Human and mouse RNAseq data analysis

**Fig. 1:**
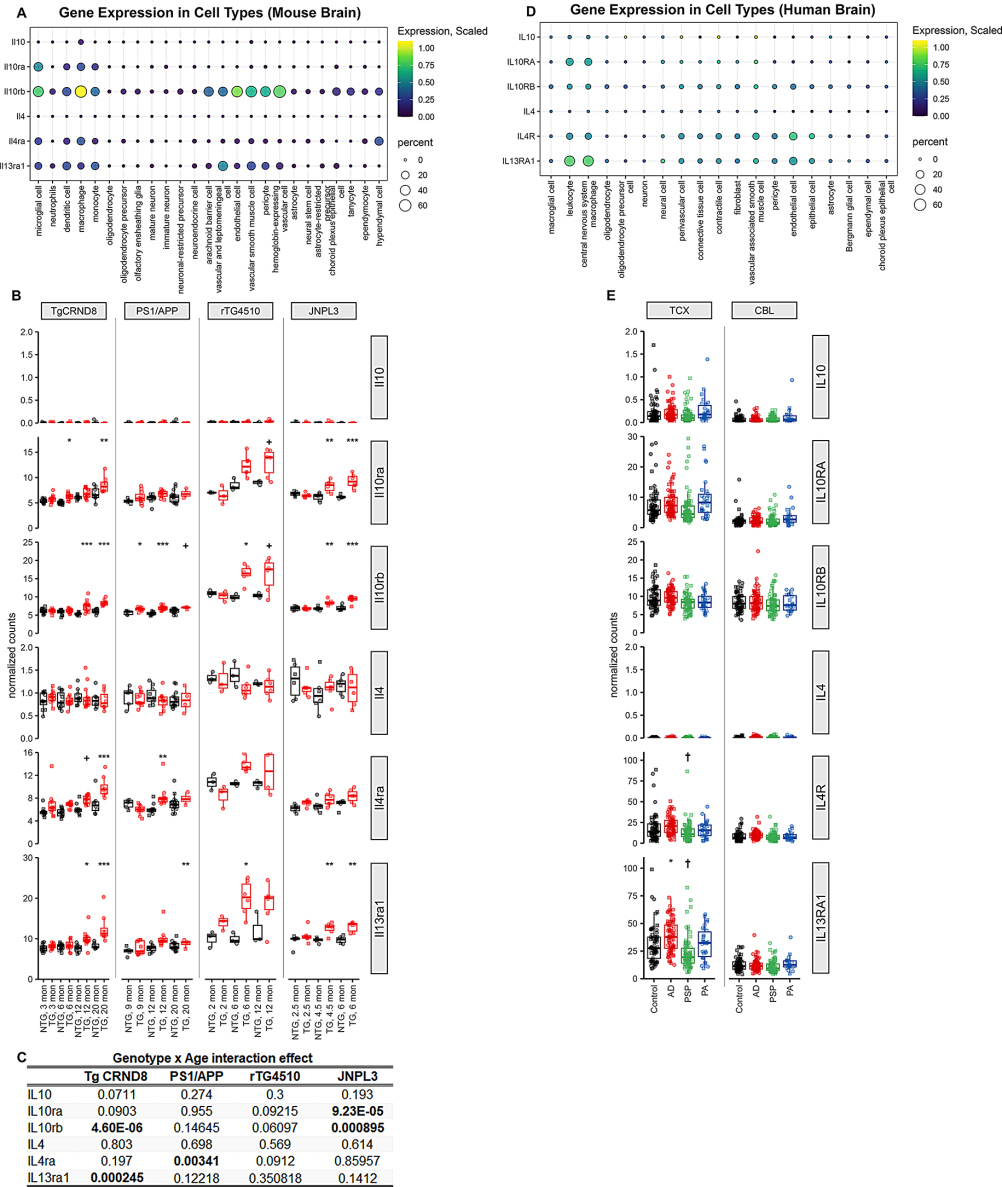
Relative expression levels of IL10 and IL4 family of ligands and receptors in preclinical mouse models of AD and AD. **A**. Normalized FPKM values of mouse transcripts obtained from different cell types, indicated on bottom, was scaled using z-score. Data was obtained from the Single Cell Portal at Broad Institute. Expression scaling denotes gene expression scaled across all cell types and percent refers to % cells within a specific cell type expressing the gene. **B**-**C**. Graphs showing normalized counts of RNA transcripts from different ages (denoted on x-axis) of *APP* TgCRND8, *APP*/*PS1 Line 85*, *MAPT* rTg4510 and *MAPT* JNPL3 mice (**B**) and statistical analysis depicting genotype by age interaction of gene expression (**C**). Each datapoint, obtained from AMP-AD synapse database, indicates an individual sample. TG, transgenic for human transgene indicated on top panel; NTG, nontransgenic wild type genetic background-matched mice. 1-way Anova, *p < 0.05, **p < 0.01, ***p < 0.001. **D**. Normalized FPKM values of human transcripts obtained in each cell type, indicated on bottom, was scaled using z-score. Expression scaling denotes gene expression scaled across all cell types and percent refers to % cells within a specific cell type expressing the gene. Data was obtained from CZ CELLxGENE Discover platform. **E**. Graphs showing normalized counts of RNA transcripts from two different human brain regions collected from Mayo AD cohorts (temporal cortex, TCX and cerebellum, CER) and available from AMP-AD synapse database. Each datapoint indicates an individual sample. 1-way Anova. Patients with different diagnoses indicated on x-axis (Control, healthy non-dementia; AD, Alzheimer’s disease; PSP, Progressive Supranuclear Palsy; PA, Pathologic Aging)

Data contained within Fig. 1 and Supplementary Fig. 1 was downloaded from the AD Knowledge Portal hosted on the Synapse website, (project ID: syn2580853) and Single Cell Portal at Broad Institute. Two-way ANOVA with interaction effect of genotype and age were calculated with the stats package version 4.4.1 in R. Graphs were generated with ggplot2 package version 3.5.1 in R.

### Statistical analysis

Data were analyzed statistically according to the methods specified in each figure legend. Data were compared by 1-way analysis of variance (ANOVA) with post-hoc Tukey’s multiple comparisons test unless mentioned otherwise in figure legend. All graphs were generated in GraphPad Prism (GraphPad Prism 9.0 Software, La Jolla, CA).

### Data availability

The datasets generated and/or analyzed during the current study are available from the corresponding authors on request. The RNAseq data described in Fig. 6 has been deposited in Synapse (Synapse ID: syn51941736).

## Results

### Rodent models of APP/Aβ and MAPT show elevated levels of Il10R and Il4R

Several lines of evidence have confirmed involvement of immune pathways in rodent models of AD, suggesting a pathologic role of inflammatory signaling in disease etiology (reviewed in [11]). We have shown earlier that contrary to expectations, Il10 and Il4, two cytokines with canonical anti-inflammatory properties, exacerbate Aβ burden in the TgCRND8 model of AD [18, 19]. To fully understand if Il10 and Il4 could play a role in these disease scenarios through canonical receptor engagement, we investigated the relative expression levels of the cytokines and receptors belonging to the Il10 and Il4 family in mice (Fig. 1A). Using single cell data available from the atlas of the aging mouse brain (https://singlecell.broadinstitute.org/single_cell/study/SCP263/aging-mouse-brain#study-visualize; [37]), we found that Il10rb is expressed in multiple cells found within the CNS, most notably microglia, leptomeningeal and arachnoid membrane cells, endothelial cells, multiple types of vascular cells and pericytes (Fig. 1A) while Il10ra is mostly limited to microglia. Il4ra shows limited presence, mainly in microglia and hypendymal cells, while Il13ra1, an alternative Il-4 receptor, is expressed by microglia and several types of vasculature and barrier-associated cells (Fig. 1A). Il10 expression assessed by deep bulk RNAseq (> 100 M reads per sample) was essentially undetectable in mice. In contrast, Il4 mRNA expression was relatively robust in mouse brain. These data suggest that in mice, Il10 signaling is likely mediated by peripheral sources of Il10 while Il4 could likely be a part of a CNS-intrinsic pathway. Analyzing mouse RNAseq data available from AMP-AD synapse database (Synapse project ID: syn2580853), we found an age-progressive increase in Il10RA, Il10RB, Il13RA1 and Il4RA in *APP* transgenic TgCRND8 mice and *MAPT* transgenic rTg4510 mice, while the levels in nontransgenic wild type mice did not vary with age (Fig. 1B). This is suggestive of a direct correlation of cytokine signaling with increasing AD-type pathologies in these mice. In the *APP*/*PS1* Line85 mice, the changes were less robust relative to age-matched wild type mice and did not show an age-dependent successive progression relative to each other (Fig. 1B). In the *MAPT* transgenic JNPL3 line, we observed age-progressive increases in these receptors, though there was variance within each cohort (Fig. 1B). A combined analysis revealed an interaction of age and genotype for Il10ra RNA in JNPL3, Il10rb RNA in TgCRND8 and JNPL3, Il4ra RNA in Line85 and Il13ra1 RNA in TgCRND8 mice (Fig. 1C). Consistent with the single cell data, RNA corresponding to the cytokines themselves were scarcely detectable in these mice, even in advanced stages of AD neuropathology.

We analyzed the single cell data available from the Human Atlas available on CZ CELLxGENE Discover platform (https://cellxgene.cziscience.com; [38]), finding that both IL10RA and IL10RB were expressed in low levels at baseline (Fig. 1D). IL4R and IL13RA1 were expressed from CNS macrophages and endothelial cells, while IL10 and IL4 cytokine levels were below detection limit (Fig. 1D). Utilizing the dataset (Synapse ID: syn5550404) derived from entorhinal cortex and cerebellum of healthy, AD, Progressive supranuclear Palsy (PSP), and ‘Pathologic Aging’ human subjects that have Aβ without dementia, we found IL13RA1 levels increased in AD patients (Fig. 1E). PSP subjects showed a lowering trend for IL4R and IL13RA1 (Fig. 1E). All other genes did not show clear disease-related patterns (Fig. 1E). Importantly, in contrast to mice, IL10 mRNA expression is variably detectable in the aged human brain, but IL4 is not. Of note, none of the transgenic mouse lines or human subjects showed appreciable levels of the other related receptors belonging to the IL10 and IL4 family– IL20RA, IL22RA1, IL22RA2, IL20RB, IL28R1/IFNLR1, IL2RG, IL13RA1 (Suppl Fig. S1).

### Expression of sIl10R in early-deposition stages attenuates Aβ plaques

**Fig. 2:**
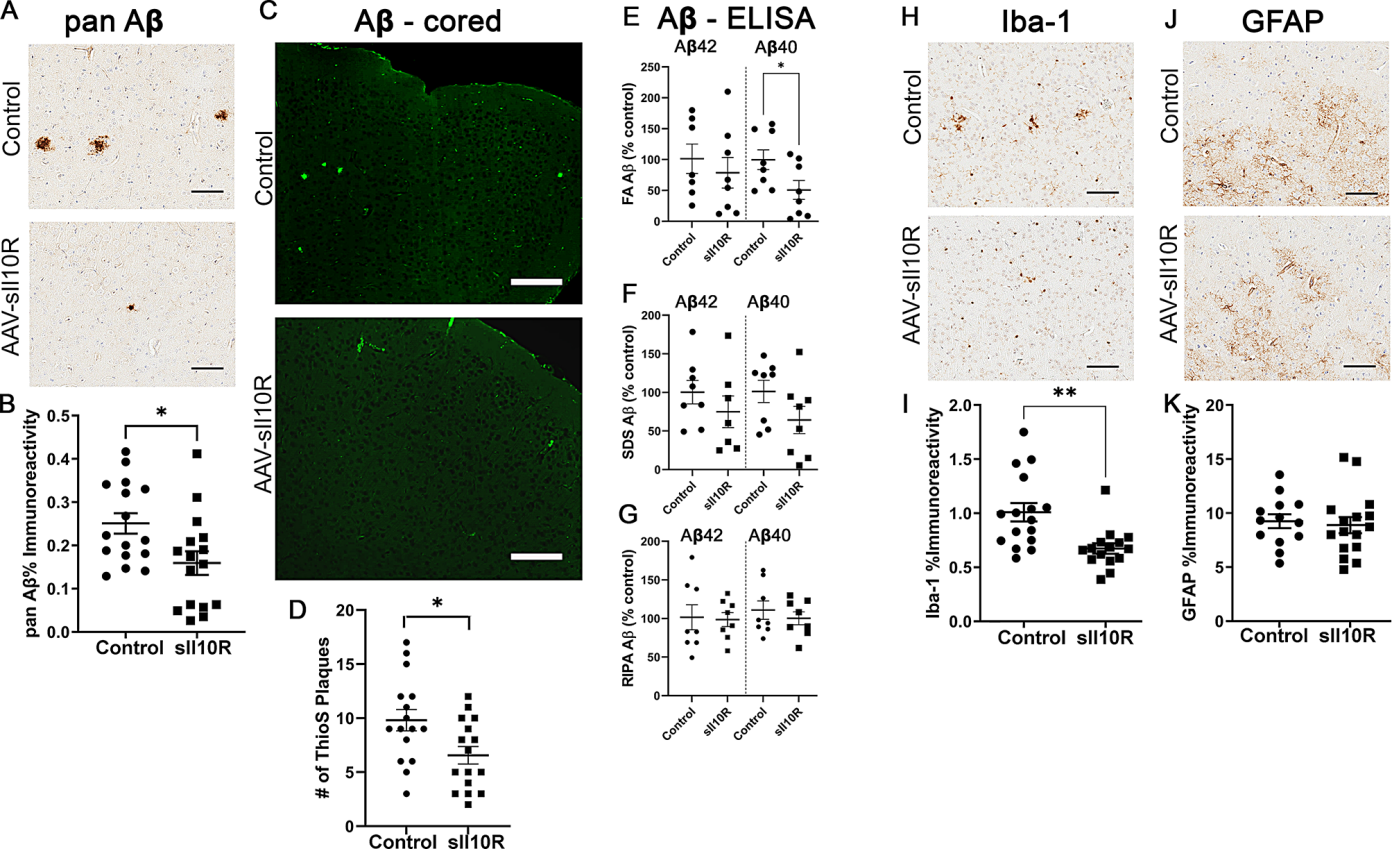
sIl10R overexpression attenuates amyloid deposition in young TgCRND8 mice. Neonatal TgCRND8 mice were injected in the cerebral ventricles with AAV-sIl10R (1E13 genomes/ml, 2 µl per ventricle) and brains were harvested at 3 months of age. Naïve age-matched mice were used as control. **A**-**D**. Representative sections showing pan-Aβ (**A**) and Thioflavin S (**C**) staining in the cortex of Tg mice. Scale, 50 μm. Quantification of the immunostaining intensity of amyloid plaques (**B**) and ThioS stained cored plaque counts (**D**) represented by mean ± standard error of the mean. Student’s two-tailed t test; *p < 0.05. E-G. FA, SDS and RIPA extracted Aβ42 and Aβ40 levels were detected by ELISA and plotted as scatter dot plot of mean ± standard error of the mean. 1-way ANOVA; *p < 0.05. *n* = 16 mice/group (**A**-**D**) and *n* = 8 mice/group (**E**-**G**). **H**-**K**. Representative sections showing Iba1 (**H**) and GFAP (**J**) staining in the cortex of TgCRND8 mice. GFAP and Iba-1 immunoreactivity burden was quantified using ImageScope analysis of staining intensity (**I**, **K**). Scale, 50 μm. Student’s two-tailed t test; **p < 0.01. *n* = 16 mice/group

Given that Il10R and Il4R increases in an age-progressive manner in TgCRND8, the pro-amyloidogenic effects of Il10 and Il4 that we previously observed in these mice could have resulted from direct receptor engagement [18, 19]. Based on this, we hypothesized that blocking these cytokine pathways using a decoy receptor strategy could reduce Aβ deposition. IL10 signaling requires the presence of heterotetramers of IL10Rα and IL10Rβ, where initial IL10 binding to its cognate site on IL10Rα subsequently allows the formation of signaling-competent heterotetramers [39]. The Il10 binding ectodomain of mouse Il10Rα was designated as soluble Il10 decoy receptor (sIl10R) and recombinant AAV1 with a V5-tagged recombinant sIl10R was delivered into neonatal TgCRND8 mice on day P2. Brains were harvested after 3-months showing high levels of protein expression and gene expression (Suppl Fig. S2A). Analysis of a set of master cytokine transcripts showed no major changes in the mice, except for lowered Tnfα (*p* = 0.0083) in the sIl10R expressing mice relative to control (Suppl Fig. S2C). Analysis of Aβ using 33.1.1 immunostaining showed robust attenuation of pan Aβ immunostaining intensity levels, compared to control mice (Fig. 2A-B; ↓36.4%, *p* < 0.05). Thioflavin S analysis in whole forebrains showed reduction in cored plaques in the sIl10R expressing mice (Fig. 2C-D; ↓33.1%, *p* < 0.05). ELISA analysis of sequentially extracted brains showed that sIl10R preferentially reduced the formic acid (FA)-associated Aβ40 levels (↓49.1%, *p* < 0.05), without affecting the Aβ42 levels (Fig. 2E). Biochemical levels of SDS and RIPA-associated soluble Aβ40 or Aβ42 were not altered in these cohorts, relative to age-matched controls (Fig. 2F-G). Using Iba-1 and GFAP immunohistochemistry, we next investigated if sIl10R-mediated attenuation in Aβ pathology was accompanied by concomitant reduction in microglial and astrocyte activation. Immunohistochemistry (Fig. 2H-I; ↓1.5x, *p* < 0.01) and immunoblotting (Suppl. Fig. S3A; ↓4.2x, *p* < 0.05) for Iba-1 revealed lower microglia numbers in sIl10R expressing mice, compared to control mice. Iba-1 immunohistochemistry analysis on non-transgenic littermates of AAV-sIl10R expressing mice did not reveal any changes in microglial burden (Suppl. Fig. S3B), indicating that the reduction was related to the Aβ phenotype. The burden of Cd68 (activated microglia) or Tmem119 (homeostatic microglia) did not differ between the sIl10R-expressing and control mice (Suppl. Fig. S3C). Analysis of astrocytes using GFAP immunohistochemistry (Fig. 2J-K) or immunoblotting (Suppl. Fig. S3D) did not reveal any changes in the sIl10R-expressing mice compared to control mice, suggesting differential involvement of sIl10R on glial cell response to Aβ deposits.

### Il10/sIl10R expression does not affect Tau pathology

**Fig. 3:**
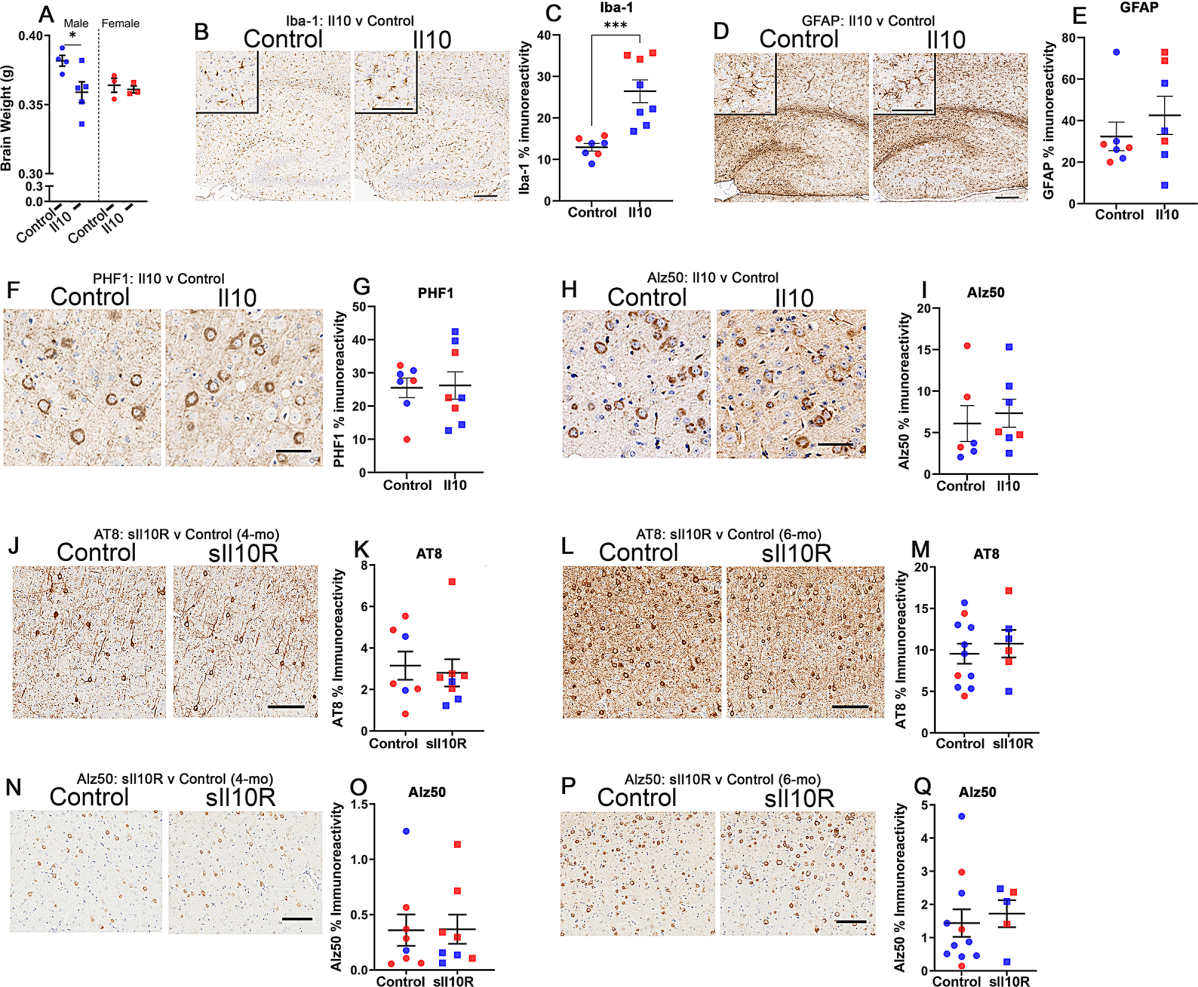
Neonatally initiated Il-10 or sIl10R expression does not alter tau pathology in rTg4510 mice. Neonatal rTg4510 mice were injected with AAV-Il10 in the cerebral ventricles and brains were harvested at 8 months of age. Naïve age-matched mice were used as control. **A**. Whole brain weights were collected immediately after euthanasia. **B**-**E**. Representative sections from male and female mice showing Iba1 (**B**), and GFAP (**D**) staining in mice brain. Scale, 50 μm (inset), 200 μm (main panel). Panel insets are zoomed depictions from cortex. Immunoreactivity was quantified using ImageScope analysis of antibody staining intensity from cortex and hippocampus and graphed (**C**, **E**). **F**-**I**. Analysis of tau pathology in Il10 expressing 8-month old rTg4510 mice. Representative sections showing PHF1 (**F**-**G**) and Alz50 (**H**-**I**) immunostaining along with corresponding immunoreactivity burden (**G**, **I**) from Il10 expressing rTg4510 mice and controls. Immunoreactivity was quantified using ImageScope analysis from cortex and hippocampus and graphed. Scale, 50 μm. *n* = 7–8 mice/group (red, female mice; blue, male mice). **J**-**Q**. Neonatal rTg4510 mice received AAV-sIl10R in their cerebral ventricles and brains were harvested at 4 months (**J**, **K**, **N**, **O**) or 6 months (**L**, **M**, **P**, **Q**) of age. Naïve age-matched mice were used as control. Representative sections showing AT8 (**J**-**M**) and Alz50 (N-Q) along with corresponding immunoreactivity burden (**K**, **M**, **O**, **Q**) from sIl10R expressing rTg4510 mice and controls. Immunoreactivity burden was quantified using ImageScope analysis from cortex and hippocampus and graphed. Scale, 50 μm. *n* = 6–11 mice/group (red, female mice; blue, male mice)

Because Il10 and sIl10R had opposing effects in TgCRND8 mice consistent with the hypothesis that Il10 worsened neuropathology, we wanted to establish the role of Il10/sIl10R signaling pathways in mouse models of tauopathy. First, we examined if expression of AAV-Il10 via neonatal delivery influenced brain pathologies in the human P301L mutant *MAPT* transgenic rTg4510 model. Intracerebral Il10 expression lowered total brain weights in 8-mo old rTg4510 male mice (Fig. 3A, ↓5.9%, *p* < 0.05). Using a targeted transcriptomic analysis, we confirmed Il10 expression in these cohorts (Suppl. Fig. S4A-B; log2Fold Change = 8.59; padj = 2.59E-30). We also noted that tau transcripts with exon10 inclusion (but not total tau RNA levels) were lowered in the Il10 expressing rTg4510 mice (Suppl. Fig. S4A-B; log2Fold change for MAPT_ex10= -0.82; padj = 0.001), which suggests that Il10 induction could be related to splicing of the tau gene. Overall, Il10 expression led to a profile associated with infection-associated immune cellular response, such as antigen processing and leukocyte-mediated cytotoxicity processes (Suppl. Fig. S4C). Expression of Il10 increased microglial gene expression, with elevated signatures consistent with both a protective homeostatic response and a neurotoxic damage-associated response (Suppl. Fig. S4D). This was also reflected in increased Iba-1 (Fig. 3B-C, ↑2.04x, *p* < 0.001) and cd11b (Suppl. Fig. S4E, ↑2.55x, *p* < 0.01) immunostaining in the Il10 expressing rTg4510 mice compared to the control mice. Astrocyte gene expression profiles (Suppl. Fig. S4D) and GFAP immunoreactivity remained unaffected (Fig. 3D-E). Analysis of tau using PHF1 (pSer396/404) antibody and conformation-sensitive MC1 and Alz50 antibodies, however, did not reveal any effect of Il10 expression on tauopathy (Fig. 3F-I; Suppl. Fig. S4F). We next tested if neonatal delivery of AAV-sIl10R would alter early tau pathogenesis or accumulation of mature tau pathology in rTg4510 mice. We analyzed tau neuropathology at 4-mo of age (prior to cortical neurodegeneration) and at 6-mo of age (when mice show cortical degeneration), finding no significant differences in either the levels of phosphorylated tau (pSer202: AT8) or misfolded tau (Alz50) between the sIl10R-expressing and control rTg4510 mice (Fig. 3J-Q). Additional immunohistochemistry analyses with Iba-1 and GFAP antibodies also did not reveal any significant changes at either timepoint in the sIl10R expressing mice relative to control mice (Suppl. Fig. S5A-H). Additionally, we also tested if neonatally initiated expression of Il10 or sIl10R would influence lifespan in homozygous JNPL3 mice, another *MAPT* transgenic rodent model expressing human mutant P301L tau. In female homozygous JNPL3 mice, neither Il10 nor sIl10R altered lifespan (Suppl. Fig. S6A) or levels of phosphorylated tau at end-stage (Suppl. Fig. S6B).

### Expression of sIl4R attenuates Aβ plaque deposition

**Fig. 4:**
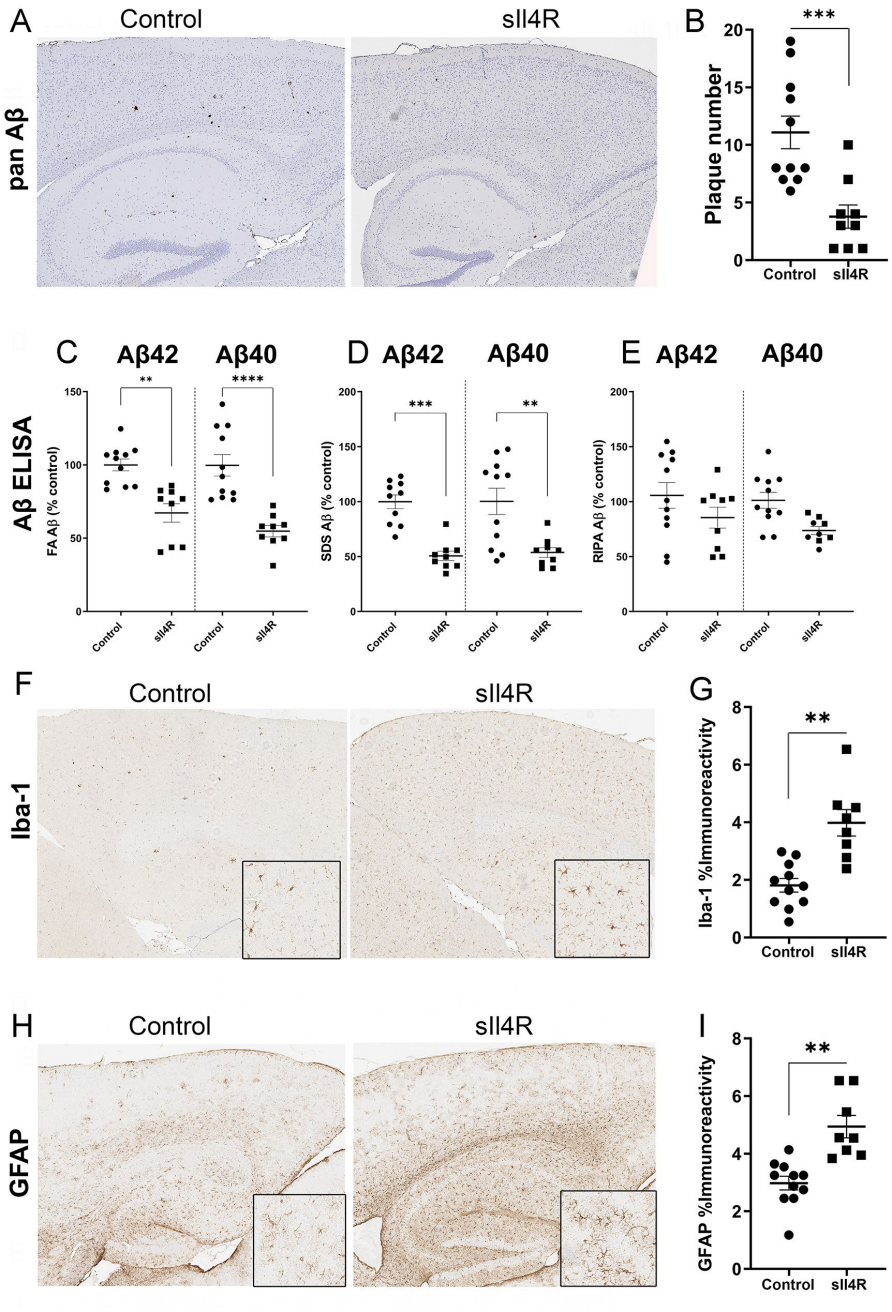
sIl4R delays amyloid deposition in young TgCRND8 mice Neonatal TgCRND8 mice were injected in the ICV with AAV-sIl4R (1E13 genomes/ml, 2 µl per ventricle) and brains were harvested at 3 months of age. Control mice were injected with saline. **A**-**B**. Representative sections showing pan-Aβ (**A**) staining in the cortex and hippocampus of Tg mice. Scale, 250 μm. Forebrain plaque count in three non-consecutive sections represented by mean ± standard error of the mean (**B**). Student’s two-tailed t test; ***p < 0.001. *n* = 11–12 mice/group. **C**-**E**. FA-, SDS- and RIPA -extracted Aβ42 and Aβ40 levels were detected by ELISA and plotted as scatter dot plot of mean ± standard error of the mean. Aβ42 and Aβ40 levels were quantified with 1-way ANOVA; *p < 0.05; **p < 0.01, ***p < 0.001, ****p < 0.0001. *n* = 11–12 mice/group. **F**-**I**. Representative sections showing Iba-1 (**F**) and GFAP (**H**) staining in the cortex and hippocampus of Tg mice. Iba-1 and GFAP immunohistochemistry burden was quantified using ImageScope analysis of staining intensity (**G**, **I**). Scale, 250 μm (main panel), 50 μm (insets). Student’s two-tailed t test; ***p < 0.001. *n* = 11–12 mice/group. Student’s two-tailed t test; **p < 0.01

IL4 is a cytokine with primarily anti-inflammatory properties whose physiological role is overlapping with and complementary to IL10 [40]. Previously, we found that AAV-Il4 expression also increased Aβ plaques [18], reminiscent of the effect of Il10 [19]. Here, we examined if a decoy receptor designed against Il4 would affect Aβ deposition. The recombinant sIl4R decoy receptor (sIl4R) containing the ligand-binding extracellular domain of full-length mouse Il4Rα was inserted into the AAV1 vector. We confirmed expression of sIl4R in the brain following neonatal delivery of recombinant AAV1 in the cerebral ventricles of TgCRND8 mice (Suppl. Fig. S2B). Analysis of a set of master cytokine transcripts showed no major changes, except for increased Il10 (*p* = 0.0215) in the sIl4R expressing mice (Suppl Fig. S2C). At 3-mo of age, examination of amyloid pathology using immunohistochemistry revealed reduction in Aβ plaque number in the cortex and hippocampus relative to age-matched controls (Fig. 4A-B; ↓65%, *p* < 0.001). This was biochemically confirmed by ELISA which showed significantly reduced levels of FA-associated Aβ42 and Aβ40 (Fig. 4C; ↓33%, *p* < 0.05 for Aβ42 and ↓45%, *p* < 0.0001 for Aβ40). Similarly, SDS-associated Aβ42 and Aβ40 were also reduced in sIl4R-expressing mice (Fig. 4D; ↓50%, *p* < 0.001 for Aβ42 and ↓47%, *p* < 0.01 for Aβ40). RIPA-soluble levels of Aβ40 and Aβ42 were not altered in the presence of sIl4R (Fig. 4E). We analyzed if sIl4R-mediated reduction in Aβ was also accompanied by a concomitant attenuation of gliosis. Unexpectedly, we observed that both microglial proliferation (Fig. 4F-G; Iba-1, ↑2.2x, *p* < 0.01) and astrocyte proliferation (Fig. 4H-I: GFAP, ↑1.6x, *p* < 0.01) increased in sIl4R-expressing TgCRND8 mice relative to age- and genotype-matched controls.Furthermore, this result was confirmed by Iba-1 immunoblotting demonstrating higher microglia numbers in sIl4R expressing mice, compared to control mice (Suppl. Fig. S7A). The burden of Cd68 (indicating activated microglia) was increased in sIl4R-expressing mice, however, the burden Tmem119 (homeostatic microglia) did not differ between the sIl4R-expressing and control mice (Suppl. Fig. S7B; Cd68, ↑1.7x, *p* < 0.05). GFAP immunoblotting showed a higher, albeit statistically insignificant, trend in the sIl4R-expressing animals (Suppl. Fig. S7C).

### Post-plaque expression of sIl10R and sIl4R has differential effects on Aβ deposition and glial proliferation

**Fig. 5:**
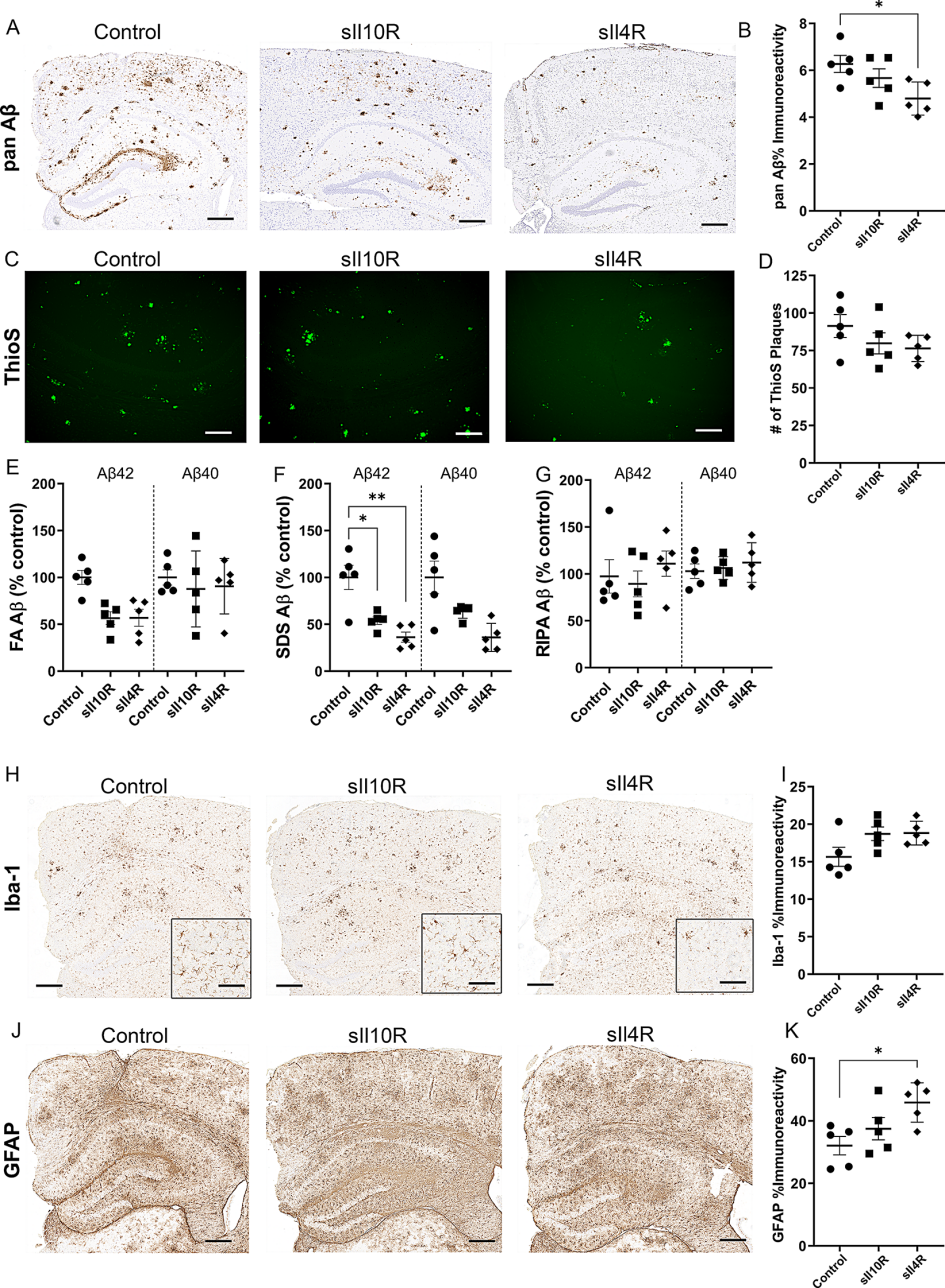
Intrahippocampal expression of sIl10R and sIl4R prevents acceleration of amyloid deposition in aged TgCRND8 mice. 6-month-old TgCRND8 mice were stereotaxically injected in the hippocampus with AAV-sIl10R and AAV-sIl4R and aged to 9 months of age. AAV-GFP was used as control. **A**-**D**. Representative sections showing pan-Aβ (**A**) and Thioflavin S cored Aβ deposits (**C**) staining. Scale, 250 μm. Quantification of the forebrain amyloid immunostaining intensity burden and Thioflavin S stained cored plaque count in three non-consecutive sections represented by mean ± standard error of the mean (**B**, **D**). 1-way ANOVA, *p < 0.001. **E**-**G**. FA-, SDS- and RIPA-extracted Aβ42 and Aβ40 levels were detected by ELISA and plotted as scatter dot plot of mean ± standard error of the mean. Aβ42 and Aβ40 levels were quantified with 1-way ANOVA; **p < 0.01, ***p < 0.001, ****p < 0.0001. **H**-**K**. Representative sections showing Iba-1 (**H**) and GFAP (**J**) staining. Scale, 250 μm (main panel), 50 μm (inset). Iba-1 and GFAP immunohistochemistry burden was quantified using ImageScope analysis (**I**, **K**). 1-way ANOVA, *p < 0.001. 1-way ANOVA; **p < 0.01. *n* = 5 mice/group

The prophylactic studies of the decoy receptors described above were conducted in separate cohorts of mice at University of Florida and Emory University. To further assess the ability of these decoy receptors to impact amyloid deposition and enable a direct head-to-head comparison in a therapeutic setting, we stereotaxically delivered AAV-sIl10R or AAV-sIl4R in the hippocampus of 6-mo old TgCRND8 mice and aged these to 9-mo. We confirmed the expression of these transgenes using immunohistochemistry (Suppl. Fig. S2D). We found that sIl10R expression modestly lowered the total hippocampal Aβ burden (↓10%, *p* = 0.2912) and Thioflavin-S cored plaque number (↓13%, *p* = 0.2982) (Fig. 5A-D). On the other hand, ELISA analysis showed that sIl10R expression preferentially reduced SDS-associated Aβ42 (↓44%, *p* = 0.158 for FA-Aβ42 and ↓47%, *p* < 0.05 for SDS-Aβ42), without altering FA-associated or SDS-associated Aβ40 levels (Fig. 5E-F). The RIPA-soluble levels of Aβ42 or Aβ40 were not affected by sIl10R expression (Fig. 5G). We did not observe any significant changes in gliosis in sIl10R expressing mice relative to controls, when examined using Iba-1 and GFAP antibody immunostaining (Fig. 5H-K). Neither Cd68-positive reactive microglia, nor Tmem119-positive homeostatic microglia burdens were altered in sIl4R and sIl10R-expressing mice relative to controls (Fig. S7D).

In the intrahippocampal cohort that was analyzed at 9-mo of age, sIl4R expression initiated in 6-mo old TgCRND8 mice led to significantly lower amyloid burden (Fig. 5A-B; ↓24%, *p* < 0.05). However, we did not observe any changes in the Thioflavin-S levels in these two groups compared to control TgCRND8 mice, indicating that cored plaque pathology was unaffected by sIl4R expression (Fig. 5C-D). There was selective reduction in SDS-associated Aβ42 relative to the control mice (Fig. 5E-F; ↓43%, *p* = 0.1654 for FA-Aβ42 and ↓64%, *p* < 0.01 for SDS-Aβ42). While the FA-soluble Aβ40 was unchanged between the two groups, SDS-associated Aβ40 levels were modestly decreased in the sIl4R expressing TgCRND8 mice (Fig. 5E-F; ↓64%, *p* > 0.99). These observations indicated that sIl4R had differential preferences for attenuation of Aβ species detected using biochemical methods that capture a broader spectrum Aβ versus immunohistochemical methods that preferentially recognize Aβ deposits. Soluble levels of Aβ42 and Aβ40 in the RIPA fraction remained unaltered in sIl4R-expressing and control TgCRND8 mice (Fig. 5G). We also examined glial proliferation using Iba-1 and GFAP immunostaining. sIl4R expression increased astrocytosis (↑1.4x, *p* < 0.01) but only modestly increased microgliosis (↑1.2x, *p* = 0.0607) when compared to the control mice (Fig. 5H-K).

### Transcriptome analysis reveals differential outcomes of decoy receptor expressing TgCRND8 mice

**Fig. 6:**
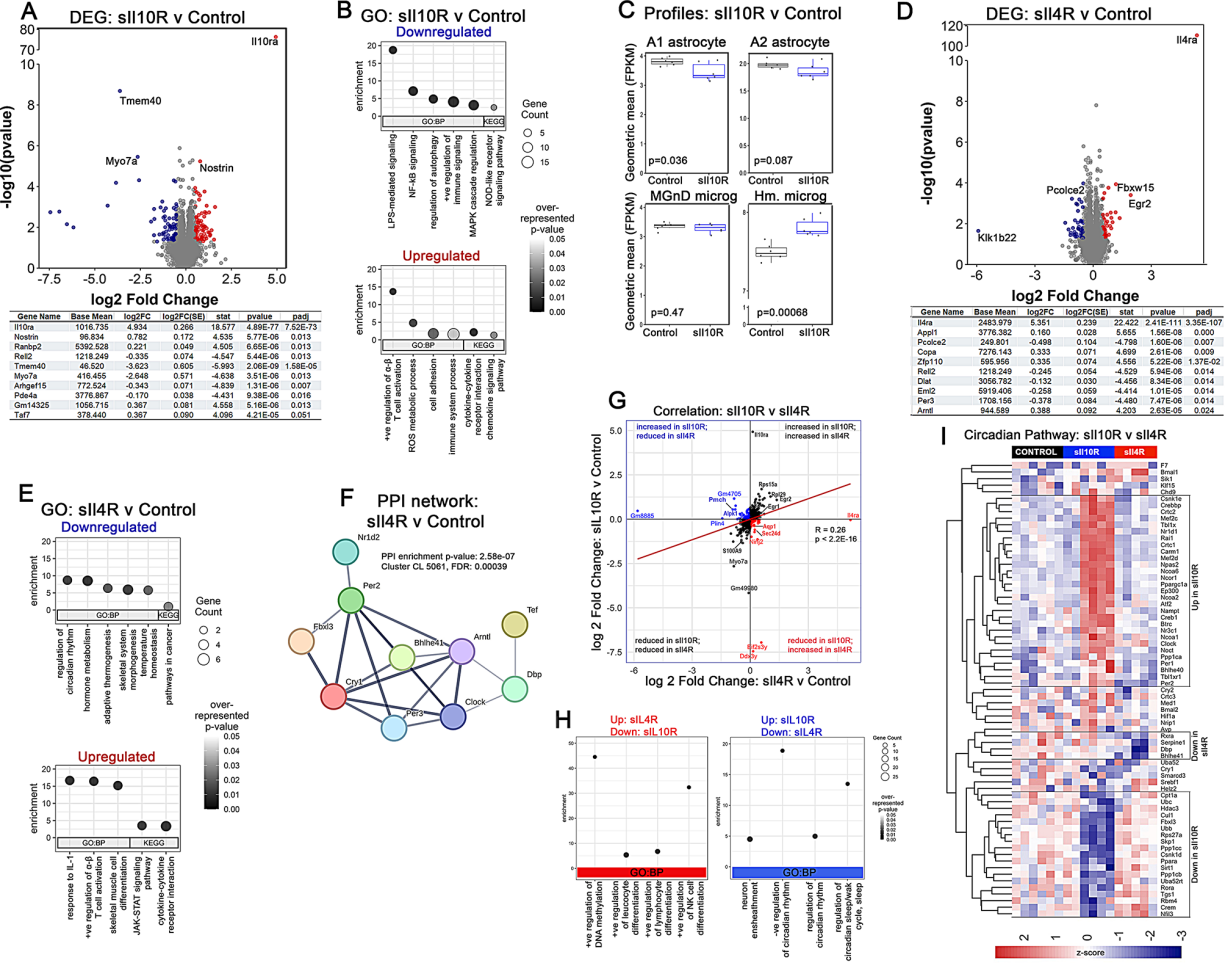
Unbiased transcriptomic analysis of TgCRND8 mice expressing sIl10R and sIl4R. **A**-**C**. Volcano plot based on fold change (red, upregulated genes, blue, downregulated genes) with list of top 10 altered genes (**A**) and GO pathways based on enriched genes (**B**) in 3-month-old TgCRND8 mice expressing sIl10R relative to control mice. padj, p-values adjusted for multiple comparison; FC, fold change. AD-associated astrocyte or microglia functional subtypes were tabulated in these mice (**C**). **D**-**E**. Volcano plot based on fold change (red, upregulated genes, blue, downregulated genes) with list of top 10 altered genes (**D**) and GO pathways based on enriched genes (**E**) in 3-month-old TgCRND8 mice expressing sIl4R relative to control mice. padj, p-values adjusted for multiple comparison; FC, fold change. **F**. PPI analysis in sIl4R expressing TgCRND8 mice using STRING revealed an enriched network of genes consistent with circadian rhythm function. *n* = 6mice/group. **G**. Correlation graph showing significantly changed genes in AAV-sIl10R and sIl4R expressing mice plotted by log2 fold change Genes with congruent changes (grey) are in the upper right (up-regulated) and lower left (down-regulated) quadrants. Unique changes in gene expression (blue) are shown in the upper left quadrant (up-regulated in sIl10R but down-regulated in sIl4R) and the lower right quadrant (down-regulated in sIl10R but up-regulated in sIl4R). Spearman’s correlation analysis was performed and results are indicated. **H**. GO-seq analysis of the genes that were differentially altered in the sIl10R and sIl4R expressing TgCRND8 mice. **I**. Differential changes in circadian pathway (Reactome) in sIl10R and sIl4R expressing TgCRND8 mice based on normalized z-score (scale depicted below). DEG, differentially expressed genes; GO, gene ontogeny; Hm, homeostatic; MGnD, neurodegenerative microglia; PPI, protein protein interaction; padj, *p*-values adjusted for multiple comparisons. *n* = 6mice/group

We used RNAseq to assess gene expression changes due to sIl10R and sIl4R expression in TgCRND8 mice relative to age- and genotyped matched controls in the neonatal cohort. We first confirmed sIl10R expression (Il10ra, log2 fold change = 4.92, padj = 4.45E-72) (Fig. 6A). In total, 1237 genes were significantly up- or down-regulated in the sIl10R cohort (*p* < 0.05), with 10 reaching statistical significance of padj < 0.05 following adjustment for multiple testing (Fig. 6A). Among the up-regulated genes that survived correction for multiple testing were Il10ra, Nostrin and Ranbp2 (Fig. 6A). Nostrin is expressed exclusively in endothelial cells and its overexpression inhibits eNOS-dependent NO production [41], suggesting attenuation of vascular oxidative stress [42]. Ranbp2 influences energy homeostasis and glycolysis pathways critical to neuronal viability [43]. Together, this would suggest that sIl10R-induced Aβ reduction mitigated stress signaling pathways. Among the down-regulated transcripts were Tmem40 and Myo7a. Both Tmem40 and Myo7a are present in mouse microglia/macrophages and absent in neurons and astrocytes (https://brainrnaseq.org), possibly consistent with reduced microgliosis in sIl10R expressing mice. Goseq analysis of the differentially expressed genes (DEG) overwhelmingly point to a mechanism involving immune pathways in these mice, especially in those pathways corresponding to down-regulated genes. Among the down regulated pathways are regulation of lipopolysaccharide-mediated signaling (BP, *p* = 5.4E-06), positive regulation of immune response (BP, *p* = 2.25E-05), regulation of autophagy (BP, *p* = 0.000378) and NOD-like receptor signaling pathway (KEGG, *p* = 0.024) (Fig. 6B). Enrichment analysis of upregulated genes identified positive regulation of α/β T cell differentiation (BP, *p* = 0.00128), reactive oxygen species metabolic process (BP, *p* = 0.0057), cell adhesion (BP, *p* = 0.013), immune system process (BP, *p* = 0.037), cytokine-cytokine receptor interaction (KEGG, *p* = 0.0006) and chemokine signaling (KEGG, *p* = 0.024) (Fig. 6B). This indicates that in the context of Aβ, sIl10R could function as an immune checkpoint inhibitor. Examination of cell-types specifically affected in the sIl10R expressing mice revealed modest changes in cell-type specific gene expression, mostly in neurons, myelinating oligodendrocytes and endothelial cells (Suppl. Fig. S3F; *p* = 0.025 for neurons, *p* = 0.045 for oligodendrocytes; *p* = 0.023 for endothelial cells), with no global alterations in astrocytic, microglial or pericyte DEGs. Assessment of different astrocyte and microglial signatures associated with neurodegenerative diseases (reviewed in [44]) showed reduction in A1 astrocyte and increased homeostatic microglia (Fig. 6C; *p* = 0.036 for A1 astrocyte; *p* = 0.00068 for homeostatic microglia). No significant changes were observed in gene expression corresponding to A2 astrocyte or AD-associated neurodegenerative MGnD microglia (Fig. 6C).

In the AAV-sIl4R cohort, the most highly altered transcript was Il4ra (log2 fold change = 5.17, padj = 2.55E-78) confirming the expression of the transgene decoy receptor (Fig. 6D). A total of 1335 genes were significantly altered, with 20 genes that survived adjustment for multiple comparisons. Il4ra, Appl1, Copa and Pcolce2 are some of the genes that were significantly altered in the sIl4 expressing TgCRND8 mice (Fig. 6D). Appl1 (Adaptor Protein, Phosphotyrosine Interacting With PH Domain And Leucine Zipper 1) is a multi-functional protein that regulates immune response and endosomal trafficking. Similarly, Copa (COPI Coat Complex Subunit Alpha) is a subunit of the coatomer that regulates intracellular protein transport from the ER to the trans Golgi network. Pcolce2, or procollagen C-endopeptidase enhancer 2, is an extracellular matrix glycoprotein involved in multiple functions, including cholesterol transport. Pathway analysis of downregulated genes that were statistically enriched in sIl4R expressing TgCRND8 (p-value < 0.05) revealed several metabolic pathways such as circadian rhythm and hormone processes (GO:0042752, *p* = 0.00372; GO:0042445, *p* = 9.73e-04), thermogenesis (GO:1990845, *p* = 0.0107) and cancer (KEGG 05200, *p* = 0.0218) were affected (Fig. 6E). Enriched processes that were upregulated belong to immune pathways, such as response to IL-1 (GO:0070555, *p* = 7.48e-04) and JAK-STAT signaling (KEGG 04630, *p* = 1.94e-03), which would be consistent with increased gliosis observed with immunohistochemistry (Fig. 6E). Protein-protein interaction analysis in the sIl4R expressing mice using STRING confirmed a circadian rhythm cluster that forms a physical sub-network (CL:5061; FDR = 0.00039) (Fig. 6F). This was related to differential expression of RNAs corresponding to clock genes in these mice, such as Per3 (padj = 0.014) and Arntl/Bmal3 (padj = 0.024) (Fig. 6D, table). We did not observe any alterations in cell type-specific DEG profiles, such as DAM, MGnD or PIG that are typically associated with neurodegenerative microglia (data not shown).

### Differential hub genes underlie the phenotypes observed in sIl10R and sIl4R expressing TgCRND8 mice

Comparing the DEGs (*p* < 0.05) in the sIl10R and sIl4R expressing cohorts, we found that the DEGs were only modestly correlated (correlation coefficient = 0.26, *p* < 2.2E-16) (Fig. 6G). While genes related to transcription (Egr1, Egr2, Rps15a, Rpl29) and myeloid signaling (S1009) are changed similarly in both groups, notable factors involved in axonal growth (Ninj2), vesicle transport and coat proteins (Sec24d, Plin4) and neurotransmitters (Pmch) are uniquely altered in the two cohorts. GO analysis of transcriptome that is downregulated in sIl10R-expressing mice but upregulated in sIl4R mice identified pathways related to DNA methylation (*p* = 3.5E-05) and regulatory pathways related to lymphocytes and leukocytes (*p* < 0.00012) (Fig. 6H). Pathways that are upregulated in sIl10R mice but downregulated in sIl4R mice identified ensheathment of neurons (*p* = 0.0003) and several pathways related to circadian rhythm and sleep (*p* < 0.001) (Fig. 6H). Regulation of circadian rhythm was also significantly enriched in DEG that were simultaneously enriched in sIl10R and sIl4R-expressing mice (*p* = 0.008) but in opposing directions, suggesting that this pathway was generally associated with decoy receptor expression. We examined the individual expression levels of the core circadian rhythm genes in the two cohorts (Reactome: R-HAS-400253), identifying specific clusters of co-expressed genes related to circadian cycle that are differentially altered in the decoy receptor expressing mice in the context of Aβ reduction (Fig. 6I). Together, this analysis reveals an unexpected involvement of circadian processes in these two treatment groups.

## Discussion

An abundance of gliosis and emergence of dysfunctional activation patterns of brain immunity characterize neurodegenerative diseases, such as AD [10, 11, 45]. Following up on previous studies demonstrating the pro-amyloidogenic effects of Il10 and Il4 [18, 19], here we tested if a decoy receptor strategy against these cytokines would mitigate AD-typical amyloid and tau pathology. We show that (a) intracerebral expression of both sIl10R and sIl4R effectively prevent Aβ deposition when present prior to plaque onset; (b) sIl10R and sIl4R have opposing effects on astrocyte and microglial proliferation in an AD-amyloidosis model; (c) sIl4R preferentially reduced Aβ when expressed after plaque onset, indicating therapeutic efficacy; (d) RNAseq analysis indicates that sIl10R and sIl4R mediated plaque reduction was associated with unique immune profiles; and (e) neither sIl10R nor Il10 had measurable effects on tau proteinopathy. Together, our data adds to the growing understanding of the concept of immunoproteostasis [11], where we posited that engagement of specific immune signaling pathways could have context-dependent outcomes, such as the extent of proteinopathy burden (prodromal vs. advanced) and the type of prevalent proteinopathy burden (Aβ vs. tau).

Decoy receptors hold promise as effective modalities in mitigating immune and metabolic disorders [46]. These are primarily receptor ectodomains that bind ligands, without being able to transduce downstream signaling, thus essentially blocking ligand signaling. One of the best-known decoy receptors used in the clinic is etanercept, a dimerized form of soluble TNF-RII conjugated to human IgG1, that can bind with high affinity to TNFα and effectively treat rheumatoid arthritis [47]. While such therapies have not yet been validated for neurodegenerative diseases in general, we had earlier established the efficacy of utilizing solubilized forms of Toll-like receptors as an anti-amyloidogenic reagent through direct binding to Aβ [32]. This current study is an extension of this alternate paradigm for AD immuno-biotherapy by utilizing naturally occurring receptor decoys for Il10 and Il4. A soluble homolog of the IL10R has been identified from placenta [48] while a natural soluble form of IL4R is produced in the serum following proteolytic shedding [49]. Because these are physiologically tolerated, they could, in theory, serve as safe anti-Aβ reagents.

Our data indicates that the soluble immune receptors targeting the Il10 and Il4 pathways reduce Aβ pathology through unique pathways. Immunohistochemistry analyses demonstrated that these soluble receptors have unique effects on microgliosis and astrocytosis, with sIl4R expression increasing microgliosis and astrocytosis, even when the Aβ burden was substantially reduced. sIl4R expression was also associated with increased transcription of JAK-STAT signaling pathway and response to Il-1. On the other hand, sIl10R reduced microglial proliferation, increased gene expression indicative of homeostatic microglia, and mitigated AD-type signaling profiles. This data from the prophylactic study points to sIl10R influencing several immune pathways, consistent with an immune checkpoint inhibitor function. Simultaneously, we observed that genes related to circadian pathways and sleep featured commonly in both groups. PPI analysis confirmed that sIl4R expression altered diurnal-related pathways, including circadian function and thermogenesis. Sleep-wake cycle is disrupted early in AD, and this desynchronization is mechanistically connected directly to Aβ levels (reviewed in [50]). Global loss of function of the clock gene Bmal1 that drives the circadian diurnal control of sleep-wake cycle increased Aβ deposition, which was not observed in CNS-specific deletion of Bmal1 [51]. Recent studies have confirmed that the molecular and functional properties of microglia, including immune gene expression and phagocytotic response, are tightly regulated in a diurnal manner by microglial clock genes [52, 53]. In our study, core circadian genes were altered in the brains of both sIl10R and sIl4R mice, albeit in different directions, raising the possibility that one of the mechanisms by which decoy receptor engagement reduces Aβ could be tied to the local circadian clock function in microglial populations.

Transcriptome data from both human subjects and AD mouse models show that while cytokine receptors increase with disease pathology, there is no change in the brain cytokine transcripts. This suggests that the Il10R and Il4R could engage with their cognate ligands that are primarily originating in the periphery. Indeed, previous data shows that microglia do not typically produce Il10 and leucocyte-derived Il10 communicates with brain-resident microglia to prevent its hyperactivation [54]. The contribution of peripherally sourced cytokines remains an important but underappreciated facet in the AD cascade [55].

In the prophylactic paradigm, where sIl10R and sIl4R expression were initiated prior to onset of Aβ plaque deposition, we observed robust attenuation in both Aβ burden as well as Thioflavin-S reactive cored Aβ plaques. In the treatment paradigm, where the decoy receptors were delivered in mice with pre-existing robust Aβ pathology, we observed that sIl4R, but not sIl10R, was able to reduce Aβ burden. Notably in this paradigm, neither decoy receptor was able to reduce the levels of Thioflavin S-reactive cored plaques, indicating that once Aβ plaques are assembled into dense-core deposits, they are resistant to being cleared.

In previous studies, we and others have observed that engagement of immune factors have opposing effects on Aβ and tau pathologies [36, 56, 57]. One unexpected outcome of this study was that IL10 expression did not trigger tau pathology, in spite of its robust effects on innate immunity (this study) and Aβ [19]. Previous experimental evidence, including transcriptomic analysis on tau transgenic and human brains, have suggested that tau etiology is connected to microglial and astrocytic activation [1, 58–60]. In particular, a recent report indicated that endotoxicosis in an Il10 deficient background induced phosphorylation of mouse tau by up-regulating neuroinflammation [58]. We observed that Il10 expression in rTg4510 mice was followed by increased microglial activation, without astrocyte proliferation being affected. Il10 also increased the expression of genes that are part of the AD/Aβ-associated dysfunctional microglial profile, such as DAM, MGnD and PIG (reviewed in [44]). However, Il10 did not accelerate tau phosphorylation or misfolding in tau transgenic mice in our study. This leads us to conjecture that in AD, presence of Aβ and tau are both required for the neurodegenerative cascade downstream of the activation of these immune phenotypes.

Though our data suggests that inhibition of intracerebral Il4 or Il10 signaling via decoy receptors could represent a novel approach to AD immune therapy, the differences in the brain expression of these cytokines between mice and humans, may present a translational dilemma. IL10 mRNA is detected in the human brain but not the mouse brain, and conversely IL4 mRNA is not detected in human brain but is detected in the mouse brain. However, given that many insights derived from the study of Il10 and IL4 signaling in mice have translated into human therapies or therapeutic strategies [61–63], it is unclear whether this difference in cytokine expression in humans is more than an interesting observation. Indeed, our data showing the sIl10R decoy alters signaling within the brain, strongly suggests that this decoy is blocking actions of peripherally-produced Il10 on the brain receptors. Indeed, we and others have measured Il10 in the brains of naïve mice [19, 54, 64] and together with the RNAseq data, it appears that Il10 is not expressed, even in the presence of long-standing pathology.

Untargeted whole-body immune manipulation, especially those that are immune activating, are almost always associated with side effects that can limit effectiveness of the therapy. For example, pharmacological blocking of Il10 using antibodies, decoy receptors or small molecules administered peripherally could carry the unintended risk of autoimmune diseases of the gut, hepatic damage in patients with chronic infection and general immunologic anergy [65]. Likewise, while monoclonal antibodies against IL4 are already in clinical trials for atopic dermatitis, concerns remain regarding its side effects on immune susceptibility following continued use [66]. On the other hand, using AAV could potentially allow for brain-targeted delivery of soluble immune receptors that restrict these signaling pathways locally [67]. To our knowledge, this is the first example of immune cytokine decoy expression in the brain, that results in a beneficial impact on an extracellular Aβ proteinopathy. We did not observe significant influence of sIl10R in progression of tau pathology or misfolding in tau transgenic mice, which indicates that sIl10R does not have detrimental effects on tau. Overall, this and our previous study [32] establish the broad functional framework of decoy receptor strategy as disease-modifying therapies in the context of AD. Surprisingly, the RNAseq data reveal relatively minor impacts on gene expression, suggesting this type of targeted delivery approach may circumvent potential toxicities. The RNAseq data showing impacts on circadian rhythm gene expression of the decoy receptor expression is intriguing. Cytokine signaling pathways have been shown to impact peripheral immune cell circadian clocks [68, 69] and both Il10 and Il4 signaling pathways have been implicated in sleep disruption in rabbits [70–73]. In addition, sickness behavior, immune responses, and circadian disruptions appear to be highly interdependent [74]. Future studies of how these, and possibly additional cytokine decoy receptors impact circadian behaviors including sleep might yield important insights into how these cytokines impact brain function in health and aging.

### Limitations

We do not provide behavioral data in this study, which potentially could inform us on the safety profile of these decoy receptors, especially their effect on brain function and immune function. Future work is also warranted to identify the cell-type specific mechanisms, especially the cross-talk of microglia and astrocyte signaling processes underlying decoy receptor mediated Aβ attenuation.

## Conclusions

The role of individual cytokine signaling pathways in the brain, especially ones that are progressively induced with advancing proteinopathy in AD, remain obscure. Here, we provide evidence that decoy receptors antagonizing intracerebral Il10 and Il4 show robust anti-Aβ effect and that such decoy receptor strategy could be effective, in both prophylactic and therapeutic administration. Given the relative differential efficacy of sIl4R over sIl10R, it remains to be seen if solubilizing other components of the Il4 signaling complex such as Il13Rα1, which is induced in both *APP* and *MAPT* transgenic mice during aging, could also have a robust anti-amyloidogenic effect. More generally, these data establish proof of concept for CNS-restricted immune decoy receptor expression as a targeted immune therapy which could be applicable across many neurologic diseases.

## Electronic supplementary material

Below is the link to the electronic supplementary material.

**Supplementary Material 1: Additional File 1: Suppl. Fig. S1**. Normalized transcript counts of IL10 and IL4 family members in brains of rodent models and human subjects. Graphs showing normalized counts of RNA transcripts from different ages (denoted on x-axis) *APP* TgCRND8, *APP*/*PS1* Line 85, *MAPT* rTg4510 and *MAPT* JNPL3 mice are shown on left 4 panels. TG, transgenic for human transgene; NTG, nontransgenic wild type genetic background-matched mice. Graphs (right 2 panels) showing normalized counts of RNA transcripts of human brains from Mayo AD cohorts (temporal cortex, TCX and cerebellum, CER). All data obtained from AD Knowledge Portal (https://adknowledgeportal.org). Patients with different diagnoses indicated on x-axis (AD, Alzheimer’s disease; PSP, Progressive Supranuclear Palsy; PA, Pathologic Aging). Each datapoint indicates an individual sample.

**Supplementary Material 2: Additional File 2: Suppl. Fig. S2**. AAV mediated sIl10R and sIl4R expression in brain. A-B. Representative immunohistochemical images of overexpression of sIl10R and sIl4R in the cortex and hippocampus from 3-mo old TgCRND8 mice was demonstrated by anti-V5 staining. sIl10R expression following neonatal injection on day P2 (A) and sIl4R expression following neonatal injection on day P0 (B). Right panels (A, B) denote the normalized RNA transcripts of Il10RA or Il4RA (in FPKM) from Control, AAV-sIl10R and sIl4R expressing TgCRND8 mice. Each dot represents an individual mouse. FPKM, Fragments Per Kilobase of transcript per Million mapped reads. C. FPKM values of cytokine transcripts in Control, AAV-sIl10R and sIl4R expressing TgCRND8 mice. Unpaired 2-tailed t test denoted by compact letter display. *p* < 0.05 for Il-10 and Tnfα cytokines in mice groups that do not share a letter; all other cytokines are not significantly different between the three mice groups and not indicated by compact letter display. *n* = 6 mice/group. 1-way Anova did not show any statistical significance. D. 6-mo old TgCRND8 mice were injected stereotaxically in the hippocampus with AAV-EGFP, AAV-sIl4R and sIl10R and analyzed for respective transgene expression at 9-months of age. Control sections were stained with anti-EGFP antibody while sIl4R and sIl10R sections were stained with anti-V5 antibody.

**Supplementary Material 3: Additional File 3: Suppl. Fig. S3**. Effect of sIl10R expression in wild type mice and TgCRND8 mice. Neonatal mice were injected with AAV-sIl10R and brains were harvested at 3 months of age. Naïve age-matched mice were used as control. (A) Immunoblot and quantitative analysis of Iba-1 (mean ± sem) from AAV-sIl10R and Control mice following normalization to housekeeping gene (β-actin). Molecular weight markers in kDa are shown on the left of each immunoblot. *n* = 6 mice/group. Unpaired 2-tailed t test, *p < 0.05. (B) Representative sections showing Iba-1 staining in the cortex and hippocampus of NTg mice. Insets from each panel is zoomed and depicted immediately in the lower panel. Scale– 250 μm, inset − 50 μm. Iba-1 immunoreactivity was quantified using ImageScope analysis and graphed. *n* = 8 mice. (C) Representative immunostaining and quantitative analysis of Cd68 and Tmem119 staining intensity from AAV-sIl10R and Control mice. *n* = 8 mice/group. Scale − 50 μm. (D) Immunoblot and quantitative analysis of GFAP (mean ± sem) from AAV-sIl10R and Control mice following normalization to housekeeping gene (actin). Molecular weight markers in kDa are shown on the left of each immunoblot. *n* = 6 mice/group. Unpaired 2-tailed t test, *p < 0.05. (E) Cell type population analyses indicating changes in astrocyte, microglia, astrocytes, neurons, oligodendrocyte (Oligodendroc), endothelial and pericyte populations in TgCRND8 mice expressing sIl10R. Gene values were derived from RNAseq FPKM values that were normalized by calculating the geometric means. *n* = 6 mice/group. Student’s t-test with p-values indicated in each panel.

**Supplementary Material 4: Additional File 4: Suppl. Fig. S4**. Effects of Il10 expression in rTg4510 mice. A-F. Neonatal rTg4510 mice were injected with AAV-Il10 and brains were harvested at 8 months of age. Volcano plot (A), list of top altered genes (based on fold change; red, upregulated genes, blue, downregulated genes) (B), GO pathways based on enriched genes (C), and cell types and profiles altered (D) in 8-month-old rTg4510 mice expressing Il10 relative to transgenic control mice. RNA data collected from NanoString custom panel and Neuroinflammation panel. padj, p-values adjusted for multiple comparison. Representative sections from male and female mice showing cd11b (E) and MC1 (F) staining in mice cortex. Immunoreactivity was quantified using ImageScope analysis from cortex and hippocampus and depicted. Scale– 250 μm, inset − 50 μm. DEG, differentially expressed genes; Hm, homeostatic; Ac, activated; DAM, damage-associated microglia; MGnD, neurodegenerative microglia; PIG, plaque-induced genes; padj, *p*-values adjusted for multiple comparisons. *n* = 7–8 mice (red, female mice; blue, male mice).

**Supplementary Material 5: Additional File 5: Suppl. Fig. S5**. Neonatally initiated sIl10R expression does not alter glial proliferation in rTg4510 mice. A-H. Neonatal rTg4510 mice were injected with AAV-sIl10R and brains were harvested at 4 months (A-D) or 6 months (E-H) of age. Naïve age-matched mice were used as control. Representative sections showing Iba-1 (A, E) and GFAP (B, F) staining in the cortex of mice. Immunoreactivity was quantified using ImageScope analysis from cortex and hippocampus and depicted in panel below. Scale, 50 μm. *n* = 6–11 mice.

**Supplementary Material 6: Additional File 6: Suppl. Fig. S6**. AAV-Il10 or AAV-sIl10R expression does not alter pathology in female JNPL3 mice. A-B. Neonatal JNPL3 mice were injected with AAV-Il10 or AAV-sIl10R and brains were harvested at onset of paralysis. Naïve age-matched mice were used as control. Kaplan Meier graph depicting lifespan of JNPL3 expressing different recombinant constructs (A). *n* = 8–20 female mice. Representative sections showing CP13 (pSer202) in three brain regions (forebrain, brainstem and spinal cord) of paralyzed JNPL3 expressing Il10 or sIl10R (B). Immunoreactivity was quantified using ImageScope analysis from cortex and hippocampus and depicted in panel below. Insets from each panel are zoomed and depicted immediately in the lower panel. Scale– 250 μm, inset − 50 μm. *n* = 4–8 mice.

**Supplementary Material 7: Additional File 7: Suppl. Fig. S7**. Effect of sIl4R expression on astrogliosis in wild type mice and TgCRND8 mice. (A) Neonatal mice TgCRND8 mice were injected with AAV-sIl4R and brains were harvested at 3 months of age. Naïve age-matched mice were used as control. Immunoblot and quantitative analysis of Iba-1 (mean ± sem) from AAV-sIl4R and Control mice following normalization to housekeeping gene GAPDH. Molecular weight markers in kDa are shown on the left of each immunoblot. *n* = 5 mice/group. Unpaired 2-tailed t test, *p < 0.05. (B) Representative immunostaining and quantitative analysis of Cd68 and Tmem119 staining intensity from AAV-sIL4R and Control mice. *n* = 5 mice/group. Scale − 50 μm. (C) Immunoblot and quantitative analysis of GFAP (mean ± sem) from AAV-sIl4R and Control mice following normalization to housekeeping gene (actin). Molecular weight markers in kDa are shown on the left of each immunoblot. *n* = 5 mice/group. Unpaired 2-tailed t test, *p < 0.05. (D) 6-mo old TgCRND8 mice were injected stereotaxically in the hippocampus with AAV-EGFP, AAV-sIl4R or sIl10R and analyzed for Cd68 and Tmem119 at 9-months of age. Representative immunohistochemical images in the vicinity of the injection site and quantitative analysis Cd68 and Tmem119 staining intensity from 9-mo old TgCRND8 mice was demonstrated by respective antibodies. *n* = 6 mice/group. 1-way Anova.

## Data Availability

The RNAseq data shown in Fig. 6 has been deposited in the AD Knowledge Portal (Synapse ID: syn51941736). All other data will be available upon reasonable request from the lead authors.

## Abbreviations

AAV: Adeno-associated virus
Aβ: Amyloidβ
AD: Alzheimer’s disease
CER: Cerebellum
DEG: Differentially expressed genes
GFAP: Glial fibrillary acidic protein
FA: Formic acid
FC: Fold change
FPKM: Fragments Per Kilobase of transcript per Million mapped reads
GO: Gene ontogeny
IBA-1: Ionized Calcium-Binding Adapter Molecule 1
ICV: Intra cerebro-ventricular
NTG: Nontransgenic for APP gene (wild type mice)
PA: Pathologic Aging
PSP: Progressive Supranuclear Palsy
RIPA: Radio-Immunoprecipitation Assay Buffer
SDS: Sodium dodecyl sulphate
sIl10R: Soluble mouse Interleukin 10 receptor
sIl4R: Soluble mouse Interleukin 4 receptor
TCX: Temporal cortex
TgCRND8 line: human mutant APP(swe/ind) transgenic mice
Padj: p-values adjusted for multiple comparison
PPI: Protein protein interaction mapping
