# Supplementary figures and images for: Antagonizing Il10 and Il4 signaling via intracerebral decoy receptor expression attenuates Aβ accumulation
Source: Acta Neuropathol Commun. 2025 Mar 7;13:51. doi: 10.1186/s40478-025-01968-3 (PMC11887169; doi:10.1186/s40478-025-01968-3)

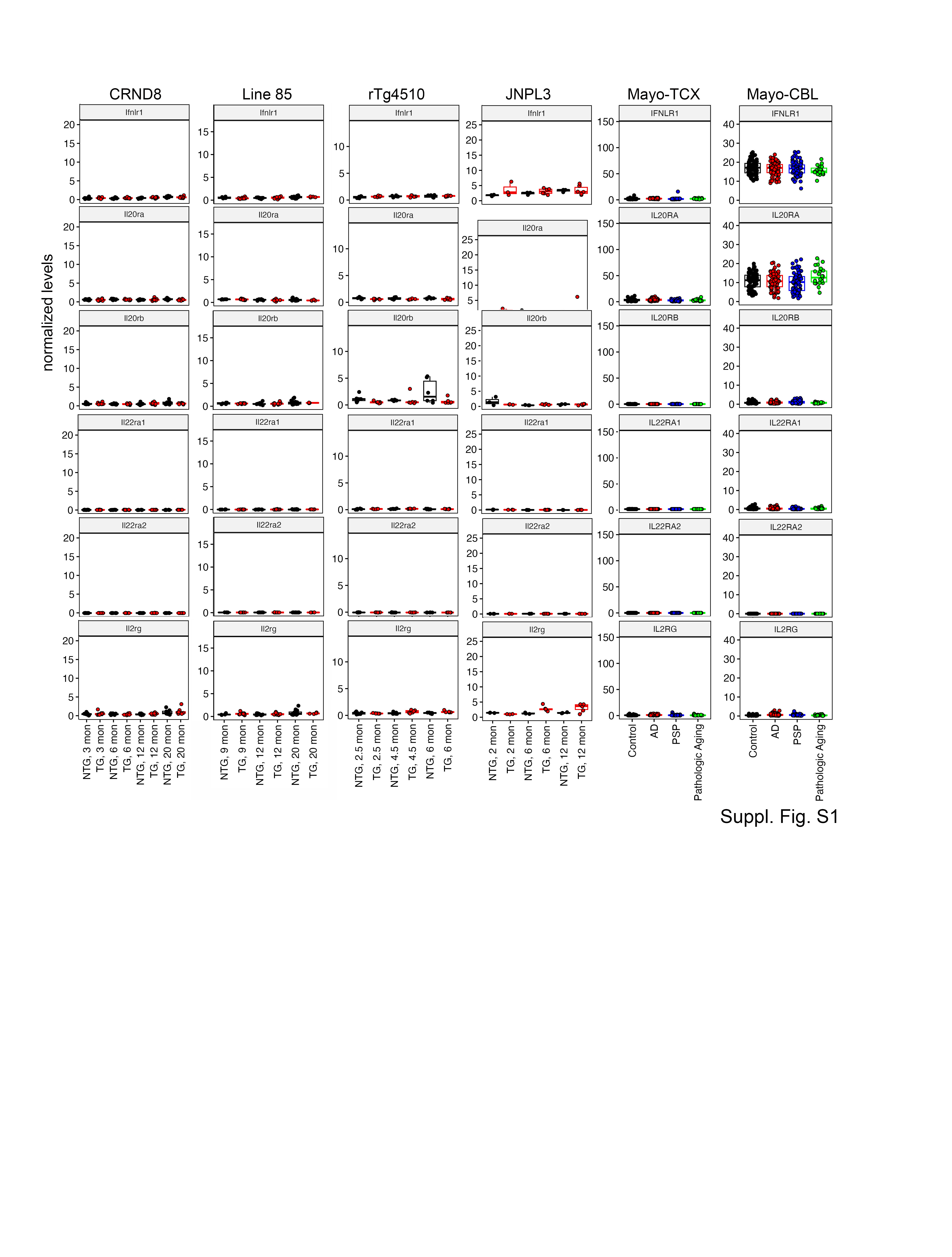

Supplement: Supplementary file 1 — Supplementary Material 1: Additional File 1: Suppl. Fig. S1. Normalized transcript counts of IL10 and IL4 family members in brains of rodent models and human subjects. Graphs showing normalized counts of RNA transcripts from different ages (denoted on x-axis) APP TgCRND8, APP/PS1 Line 85, MAPT rTg4510 and MAPT JNPL3 mice are shown on left 4 panels. TG, transgenic for human transgene; NTG, nontransgenic wild type genetic background-matched mice. Graphs (right 2 panels) showing normalized counts of RNA transcripts of human brains from Mayo AD cohorts (temporal cortex, TCX and cerebellum, CER). All data obtained from AD Knowledge Portal (https://adknowledgeportal.org). Patients with different diagnoses indicated on x-axis (AD, Alzheimer’s disease; PSP, Progressive Supranuclear Palsy; PA, Pathologic Aging). Each datapoint indicates an individual sample. [file 40478_2025_1968_MOESM1_ESM.tif]

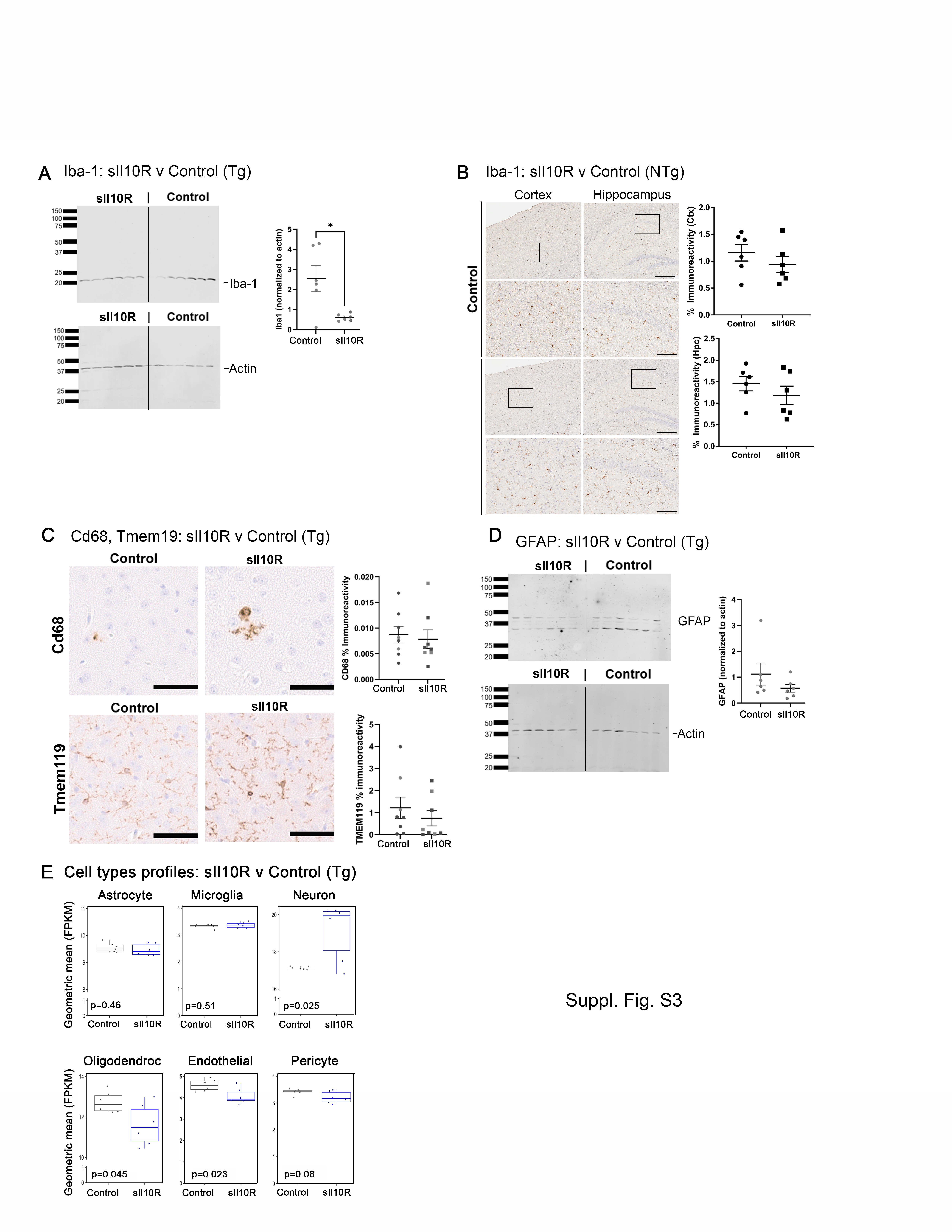

Supplement: Supplementary file 3 — Supplementary Material 3: Additional File 3: Suppl. Fig. S3. Effect of sIl10R expression in wild type mice and TgCRND8 mice. Neonatal mice were injected with AAV-sIl10R and brains were harvested at 3 months of age. Naïve age-matched mice were used as control. (A) Immunoblot and quantitative analysis of Iba-1 (mean ± sem) from AAV-sIl10R and Control mice following normalization to housekeeping gene (β-actin). Molecular weight markers in kDa are shown on the left of each immunoblot. n = 6 mice/group. Unpaired 2-tailed t test, *p < 0.05. (B) Representative sections showing Iba-1 staining in the cortex and hippocampus of NTg mice. Insets from each panel is zoomed and depicted immediately in the lower panel. Scale– 250 μm, inset − 50 μm. Iba-1 immunoreactivity was quantified using ImageScope analysis and graphed. n = 8 mice. (C) Representative immunostaining and quantitative analysis of Cd68 and Tmem119 staining intensity from AAV-sIl10R and Control mice. n = 8 mice/group. Scale − 50 μm. (D) Immunoblot and quantitative analysis of GFAP (mean ± sem) from AAV-sIl10R and Control mice following normalization to housekeeping gene (actin). Molecular weight markers in kDa are shown on the left of each immunoblot. n = 6 mice/group. Unpaired 2-tailed t test, *p < 0.05. (E) Cell type population analyses indicating changes in astrocyte, microglia, astrocytes, neurons, oligodendrocyte (Oligodendroc), endothelial and pericyte populations in TgCRND8 mice expressing sIl10R. Gene values were derived from RNAseq FPKM values that were normalized by calculating the geometric means. n = 6 mice/group. Student’s t-test with p-values indicated in each panel. [file 40478_2025_1968_MOESM3_ESM.tif]

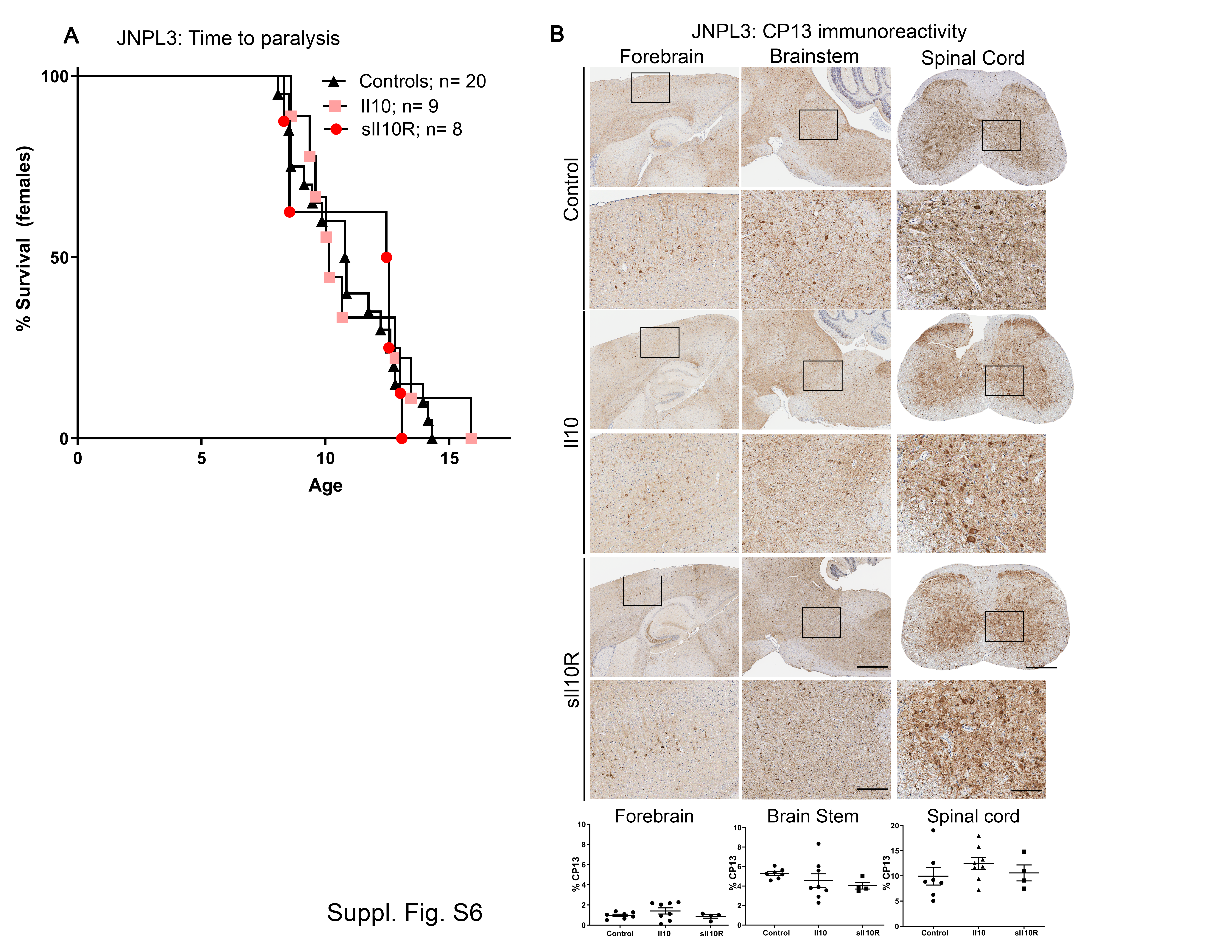

Supplement: Supplementary file 6 — Supplementary Material 6: Additional File 6: Suppl. Fig. S6. AAV-Il10 or AAV-sIl10R expression does not alter pathology in female JNPL3 mice. A-B. Neonatal JNPL3 mice were injected with AAV-Il10 or AAV-sIl10R and brains were harvested at onset of paralysis. Naïve age-matched mice were used as control. Kaplan Meier graph depicting lifespan of JNPL3 expressing different recombinant constructs (A). n = 8–20 female mice. Representative sections showing CP13 (pSer202) in three brain regions (forebrain, brainstem and spinal cord) of paralyzed JNPL3 expressing Il10 or sIl10R (B). Immunoreactivity was quantified using ImageScope analysis from cortex and hippocampus and depicted in panel below. Insets from each panel are zoomed and depicted immediately in the lower panel. Scale– 250 μm, inset − 50 μm. n = 4–8 mice. [file 40478_2025_1968_MOESM6_ESM.tif]

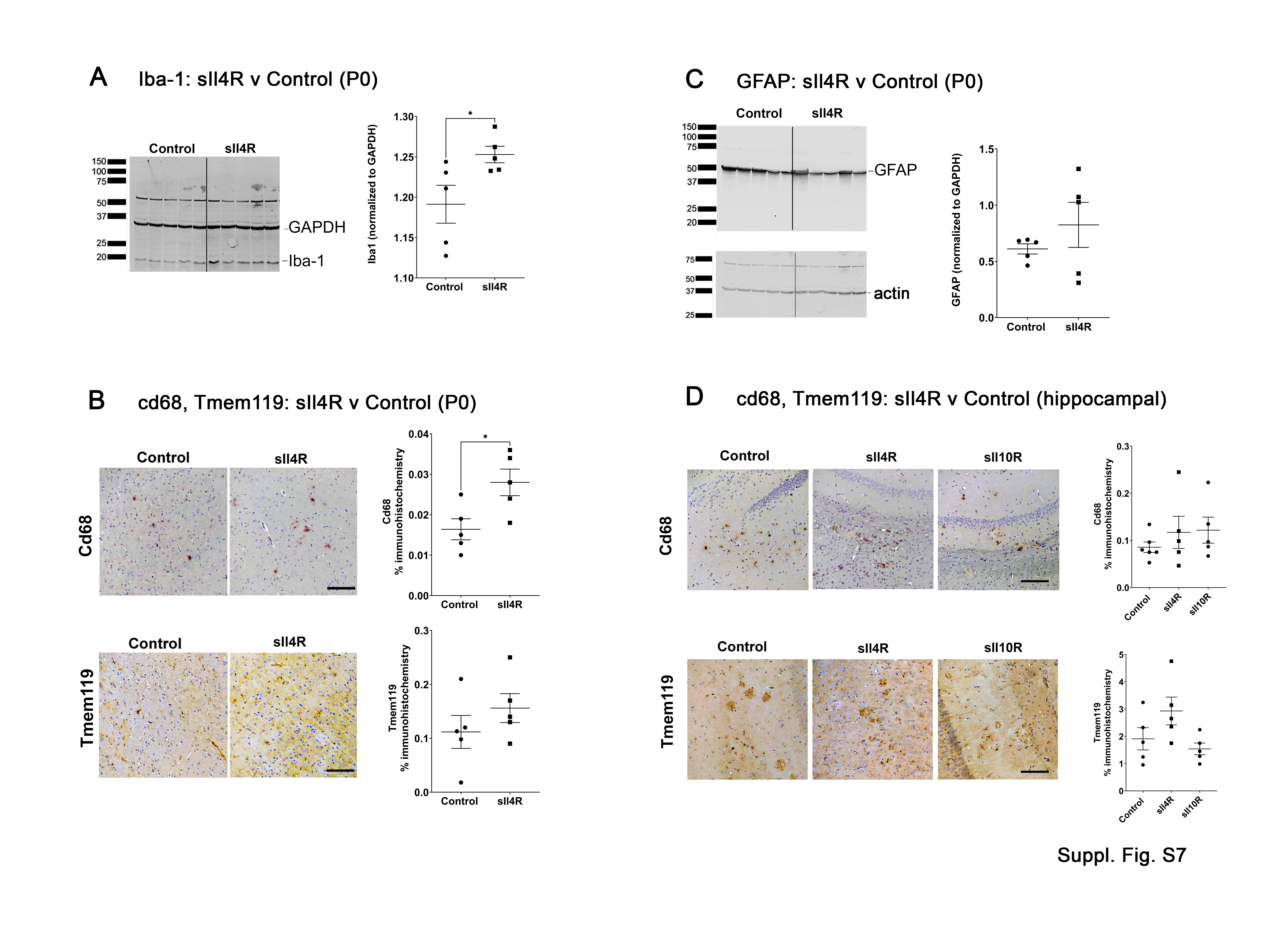

Supplement: Supplementary file 7 — Supplementary Material 7: Additional File 7: Suppl. Fig. S7. Effect of sIl4R expression on astrogliosis in wild type mice and TgCRND8 mice. (A) Neonatal mice TgCRND8 mice were injected with AAV-sIl4R and brains were harvested at 3 months of age. Naïve age-matched mice were used as control. Immunoblot and quantitative analysis of Iba-1 (mean ± sem) from AAV-sIl4R and Control mice following normalization to housekeeping gene GAPDH. Molecular weight markers in kDa are shown on the left of each immunoblot. n = 5 mice/group. Unpaired 2-tailed t test, *p < 0.05. (B) Representative immunostaining and quantitative analysis of Cd68 and Tmem119 staining intensity from AAV-sIL4R and Control mice. n = 5 mice/group. Scale − 50 μm. (C) Immunoblot and quantitative analysis of GFAP (mean ± sem) from AAV-sIl4R and Control mice following normalization to housekeeping gene (actin). Molecular weight markers in kDa are shown on the left of each immunoblot. n = 5 mice/group. Unpaired 2-tailed t test, *p < 0.05. (D) 6-mo old TgCRND8 mice were injected stereotaxically in the hippocampus with AAV-EGFP, AAV-sIl4R or sIl10R and analyzed for Cd68 and Tmem119 at 9-months of age. Representative immunohistochemical images in the vicinity of the injection site and quantitative analysis Cd68 and Tmem119 staining intensity from 9-mo old TgCRND8 mice was demonstrated by respective antibodies. n = 6 mice/group. 1-way Anova. [file 40478_2025_1968_MOESM7_ESM.jpg]
